# Microorganism-Based Larval Diets Affect Mosquito Development, Size and Nutritional Reserves in the Yellow Fever Mosquito *Aedes aegypti* (Diptera: Culicidae)

**DOI:** 10.3389/fphys.2019.00152

**Published:** 2019-04-09

**Authors:** Raquel Santos Souza, Flavia Virginio, Thaís Irene Souza Riback, Lincoln Suesdek, José Bonomi Barufi, Fernando Ariel Genta

**Affiliations:** ^1^Laboratório de Bioquímica e Fisiologia de Insetos, Instituto Oswaldo Cruz, FIOCRUZ, Rio de Janeiro, Brazil; ^2^Laboratório Especial de Coleções Zoológicas, Instituto Butantan, São Paulo, Brazil; ^3^World Mosquito Program, Rio de Janeiro, Brazil; ^4^Laboratório de Parasitologia, Instituto Butantan, São Paulo, Brazil; ^5^Instituto de Medicina Tropical de São Paulo, Universidade de São Paulo, São Paulo, Brazil; ^6^Laboratório de Ficologia, Departamento de Botânica, Centro de Ciências Biológicas, Universidade Federal de Santa Catarina, Florianópolis, Brazil; ^7^Instituto Nacional de Ciência e Tecnologia em Entomologia Molecular, Rio de Janeiro, Brazil

**Keywords:** *Aedes aegypti*, microorganism, development, nutritional reserves, digestion, yeast, bacteria, algae

## Abstract

**Background:**

Mosquito larvae feed on organic detritus from the environment, particularly microorganisms comprising bacteria, protozoa, and algae as well as crustaceans, plant debris, and insect exuviae. Little attention has been paid to nutritional studies in *Aedes aegypti* larvae.

**Objectives:**

We investigated the effects of yeast, bacteria and microalgae diets on larval development, pupation time, adult size, emergence, survivorship, lifespan, and wing morphology.

**Materials and Methods:**

Microorganisms (or Tetramin^®^ as control) were offered as the only source of food to recently hatched first instar larvae and their development was followed until the adult stage. Protein, carbohydrate, glycogen, and lipid were analyzed in single larvae to correlate energetic reserve accumulation by larva with the developmental rates and nutritional content observed. FITC-labeled microorganisms were offered to fourth instar larvae, and its ingestion was recorded by fluorescence microscopy and quantitation.

**Results and Discussion:**

Immature stages developed in all diets, however, larvae fed with bacteria and microalgae showed a severe delay in development rates, pupation time, adult emergence and low survivorship. Adult males emerged earlier as expected and had longer survival than females. Diets with better nutritional quality resulted in adults with bigger wings. *Asaia* sp. and *Escherichia coli* resulted in better nutrition and developmental parameters and seemed to be the best bacterial candidates to future studies using symbiont-based control. The diet quality was measured and presented different protein and carbohydrate amounts. Bacteria had the lowest protein and carbohydrate rates, yeasts had the highest carbohydrate amount and microalgae showed the highest protein content. Larvae fed with microalgae seem not to be able to process and store these diets properly. Larvae were shown to be able to process yeast cells and store their energetic components efficiently.

**Conclusion:**

Together, our results point that *Ae. aegypti* larvae show high plasticity to feed, being able to develop under different microorganism-based diets. The important role of *Ae. aegypti* in the spread of infectious diseases requires further biological studies in order to understand the vector physiology and thus to manage the larval natural breeding sites aiming a better mosquito control.

## Introduction

Mosquitoes are medically the most significant group of insects due to their important role in the widespread of several human infectious diseases including malaria, dengue fever, encephalitis, yellow fever and filariasis (Weaver and Reisen, 2010). The global magnitude of morbidity and mortality caused by arthropod-borne diseases has been a public health emergency of international concern. Early stages of mosquito development are related to aquatic environments, thus understanding the ecological factors involved in the aquatic habitats is essential in order to develop and improve effective strategies of mosquito control.

The biotic and abiotic environmental conditions experienced during the immature stage are determinant for the growth and development of mosquitoes. A considerable number of studies in the early 20th century devoted attention to investigating the food requirements of larvae in order to reduce or eliminate the nutritional supply of these insects in nature (Hinman, 1930). Studies on holometabolous insects suggest that well-nourished larvae become healthier adults (Zeller and Koella, 2016). The biomass accumulation of mosquitoes can be attributed to the efficiency of foraging by larvae and withstanding of starvation (Barrera, 1996). Quantitative and qualitative aspects of larval nutrition exert immediate effects on immature survivorship and development rate, which can alter population dynamics of mosquitoes and determine adults life traits (Subra and Mouchet, 1984; Gimnig et al., 2002; Barrera et al., 2006; Araújo et al., 2012; Radchuk et al., 2013; Kivuyo et al., 2014; Li et al., 2014).

Mosquito populations that develop in containers can be regulated by the availability and amount of food resources in the aquatic habitat (Washburn, 1995). Food deprivation can have several carry-over effects on mosquito life. A longer development time under conditions of food insufficiency has been observed before (Tun-Lin et al., 2000; Arrivillaga and Barrera, 2004; Dominic et al., 2005; Vantaux et al., 2016; Aznar et al., 2018), with mosquito larva that take longer time to reach pupa stage (Telang et al., 2007; Levi et al., 2014; Banerjee et al., 2015). An extended larval stage is generally associated with an increased risk of mortality as a consequence of predation, breeding site instability and human interference (Padmanabha et al., 2011). Beyond development time, the amount of food influences characteristics such as: nutritional reserves (Van Handel and Day, 1989; Briegel, 1990b; Gullan and Cranston, 1999; Arrivillaga and Barrera, 2004), adult emergence (Okech et al., 2007), body size (Grimstad and Walker, 1991; Strickman and Kittayapong, 2003; Jirakanjanakit et al., 2007; Foster et al., 2012; Aznar et al., 2018), response to repellents and insecticides (Xue et al., 1995; Xue and Bernard, 1996), survival (Landry et al., 1988; Dominic and Das, 1996; Sumanochitrapon et al., 1998; Aznar et al., 2018), sexual maturity, fecundity, egg production and longevity of the adult female (Briegel, 1990b; Nasci and Michell, 1994; Naksathit and Scott, 1998; Sumanochitrapon et al., 1998; Reiskind and Lounibos, 2009; Alto et al., 2012; Foster et al., 2012; Takken et al., 2013). The vector competence also could be influenced by the available food resource. Adults that emerge from larvae with low nutritional reserve are smaller (Lehmann et al., 2006) and require more blood feeds to produce eggs (Briegel, 1990b), which may lead to an increase in their vectorial capacity (Muturi et al., 2011). Restricted larval food can extend the time for mosquitoes to become infectious (Shapiro et al., 2016; Vantaux et al., 2016), modulate microbiota (Linenberg et al., 2016) and permissiveness to parasites (Takken et al., 2013; Linenberg et al., 2016), affecting immune traits (Suwanchaichinda and Paskewitz, 1998; Telang et al., 2012).

Previous studies reported that *Aedes aegypti* size is vulnerable to food amount and population density in immature stages (Jirakanjanakit et al., 2007). Direct measurement of the mosquito body is not satisfactory estimation of size, due to the variation of three-dimensional structures, besides the variation in the dryness of the abdomen. Weight is also an unreliable estimator of body size as it can be influenced by the blood feeding, egg production, among other factors. The mosquito body size may be adequately estimated using the wing length, or even better, using wing centroid size, an isometric and comprehensive estimator of body size (Bookstein, 1992; Siegel et al., 1992; Lounibos, 1994; Carron, 2007; Jirakanjanakit et al., 2007; Strickman and Kittayapong, 2003; Lehmann et al., 2006).

Immature stages of culicids are generally undemanding and have a pliant food behavior, ingesting through different feeding modes (e.g., filtering, suspension feeding, browsing, and interfacial feeding) organic particles in the water and almost everything available in the natural or artificial environments (Walker et al., 1988; Merritt et al., 1992; Clements, 2000). Particulate microorganisms and organic debris are commonly the main nutritional source of mosquito larvae. Bacteria, viruses, protozoa, fungi (Timmermann and Briegel, 1996; Forattini, 2002) and algae (Merritt et al., 1992; Kivuyo et al., 2014) are some of the organisms that actively contribute to foraging and development during the larval stage. Bacteria seems the most abundant microorganisms present in the larval diet, and may even be the only nutritional source for insect growth and development (Merritt et al., 1992). Pollen particles dispersed in the aquatic environment can also be used as food sources by immature forms (Ye-Ebiyo et al., 2003; Kivuyo et al., 2014; Asmare et al., 2017).

The evolutionary success and extensive dispersal of mosquitoes may have been widely motivated by symbiotic relationships with microorganisms (Ricci et al., 2011a; Coon et al., 2014). Insects harbor numerous symbiont microbial communities, which possibly supplant the number of the cells of the invertebrate (Gusmão et al., 2010). Intracellular symbionts can occur in up to 70% of all insect species, and the intestinal compartment concentrates most of these microorganisms (Gusmão et al., 2010). The contribution of the intestinal microbiota of insects in nutritional ecology is quite relevant due to their impressive biosynthetic and degradative capacity (Douglas, 2009; Kukutla et al., 2014). The insect microbiota plays an important role in the synthesis of vitamins and essential amino acids, steroids and carbohydrates metabolism and promoting the growth and development using the insulin pathway (Shin et al., 2011; Storelli et al., 2011; Douglas, 2014). Besides nutrition, symbionts aid in nitrogen fixation, behavior, reproduction, development and enhance or suppress infections by pathogens (Dillon and Dillon, 2004; Hegde et al., 2015).

Aspects such as digestion, processing, absorption and detoxification of such generalist diets are the result of refined interactions with symbionts and digestive enzymes (Fisk and Shambaugh, 1952; Geering and Freyvogel, 1975; Marinotti and James, 1990; Ho et al., 1992; Souza et al., 2016). It is still unclear as the several microbial nutritional sources may influence the physiology of larval mosquito and which are the main enzymes involved in the digestion of these nutrients.

In this study, we investigated *Ae. aegypti* larval feeding using a range of microorganisms as a nutritional source. Life parameters including development rates, survival, sex ratio, body size, ingestion rates, quantity and quality of food and nutritional reserve accumulation were reported in this paper. The results suggest that microorganism-based diets can be supported by these insects in laboratory conditions and aim to provide information to laboratory breeding or studies for potential biological larvicides.

## Materials and Methods

### Mosquito Rearing

The *Ae. aegypti* specimens eggs used for this study, were originated from eggs of Rockefeller strain gently ceded by Dr. José Bento Pereira Lima - from colonies of the Laboratory of Physiology and Control of Arthropod Vectors (LAFICAVE, -IOC/-FIOCRUZ; Dr. José Bento Pereira Lima). Insects were reared until the adult stage in the Laboratory of Insect Biochemistry and Physiology (LABFISI, IOC/FIOCRUZ) under standard conditions (temperature 26 ± 1°C, relative humidity 80 ± 5% and photoperiod 12:12 h [L: D]). Newly hatched larvae derived from the same egg batch within 2 h of eclosion were fed Tetramin^®^ sprinkled on the distilled water surface until the nutritional trials being performed.

### Screening of Microorganisms

The nutritional physiology experiments were performed based on the follow microorganisms: *Serratia marcescens* (SM365), *Escherichia coli* (D31) and *Staphylococcus aureus* isolated and cryopreserved in the LABFISI, *Saccharomyces cerevisiae* (S14) kindly donated by Dr. Pedro Soares de Araújo (Chemistry Institute, University of São Paulo), *Asaia* sp. (A1), *Ochrobactrum intermedium* (Om17), *Bacillus* sp. and *Pseudozyma* sp. (Pa1) by Dr. Rod J. Dillon (Faculty of Health and Medicine, Lancaster University, United Kingdom), *Arthrospira platensis* (*Spirulina*) and *Chlorella* sp. by Dr. José Bonomi Barufi (Laboratory of Phycology, Federal University of Santa Catarina, Brazil).

### Preparation of Microorganisms Diets

Aliquots of *S. marcescens*, *E. coli*, *Bacillus* sp, *O. intermedium*, and *S. aureus* were inoculated in Luria-Bertani agar plates (LB) and incubated overnight for 24 h at 30°C. *S. cerevisiae* and *Pseudozyma* sp. were inoculated in YEPD agar plates (1% yeast extract, 2% peptone, 2% glucose/dextrose, 2% agar). Growth conditions: *S. cerevisiae* overnight for 24 h at 30°C and *Pseudozyma* sp. 48 h at 30°C. *Asaia* sp. were inoculated on GCA agar plates (2% glucose, 0.8% yeast extract, 0.7% CaCO_3_, 2% agar) and incubated overnight for 72 h at 26°C (Sant’Anna et al., 2014). Bacteria single colonies were transferred to LB medium, yeast-like fungus to YPD medium and *Asaia* sp. to GLY medium (glycerol 25 g/l, yeast extract 10 g/l, pH 5.0) in 50 mL polypropylene tubes. All strains were grown according to the incubation temperatures of each strain in a shaking incubator (150 rpm). The microbial suspensions were centrifuged (20 min, 21,000 *g*, 4°C) and the supernatant was discarded to fresh mass (FM) measurements. Cells harvested by brief spin were washed with sterile PBS three times, and finally, the bacterial and yeast pellet was resuspended in sterile water and adjusted in a concentration of 800 mg/80 mL (w/v) per strain. *Chlorella* sp. were inoculated in Bold’s Basal Medium (BBM), and *A. platensis* were inoculated in Spirulina Medium Modified (Andersen, 2005). *Chlorella* sp. and *A. platensis* were incubated at 21°C with a photoperiod of 12:12 h [L: D] in a shaking incubator (100 rpm). They were centrifuged gently (5 min, 5,000 *g*), before the measure of their biomass. Cells harvested by brief centrifugation were resuspended in their respective medium and were adjusted in a concentration of 150 mg/15 mL (w/v).

### Microorganisms Viability Trials

To evaluate the capacity of microorganisms used in this study remains alive in the aquatic environment, we observed the viability of these strains on water. Bacteria and yeast were inoculated in 50 mL polypropylene tubes in specific liquid media and growth conditions were described previously (see details in Preparation of Microorganisms Diets). Cells harvested by centrifugation (20 min, 21,000 *g*, 4°C) were resuspended in liquid media or sterile water. Twenty microliter aliquots of each suspension were placed on agar plates and the number of colonies forming units, CFU were recorded after 0, 24, 48, and 120 h. Five biological experiments were performed for statistical analysis.

### Experimental Nutrition Protocol

Ten diets were compared. Groups of 150 first-instar larvae (L1) were manually counted and transferred to each of three sterile borosilicate glass recipients (22.5 cm × 12.8 cm × 3.59 cm). Under sterile conditions, each container was filled with QSP 250 mL of sterile water or distilled water (density = 0.22 larvae/cm^2^ of surface area; depth of 39 mm). The same larval density was used in all experiments. The dietary supply was administered only at the L1, 80 mL (corresponding to 800 mg [w/v] and 16 mg/larva) of yeasts and bacterial suspensions and 15 mL (corresponding to 150 mg [w/v] and 3 mg/larva) of microalgae cultures were added to glass recipients. A slurry by mixing the components of Tetramin^®^ in distilled water was prepared to fed standard group. We used 800 mg of Tetramin^®^ resuspended in distilled water QSP to a final volume of 250 mL. Evaporated water was replaced as needed to maintain the initial volume. Three replicates were performed for each dietary experiment.

### Effects of Diets on Development and Survivorship of *Ae. aegypti*

For comparison of diet effects, developmental rate and survivorship from eclosion to adult emergence were measured. Larvae were observed daily until pupation, and dead larvae and exuviae were removed. Pupae were collected daily, counted and transferred individually into 15 mL polypropylene tubes covered with mosquito netting and filled with 4 mL of breeding water until adult emergence. The number and the sex of adults emerged were determined. Adults received only cotton wool moistened with distilled water *ad libitum*. The median time in days for pupation, the emergence of adults (males and females) and adult survivorship were calculated using the number of individuals that reached pupae or the adult stage. We also recorded the proportion of larvae that survived from L1 to the pupal stage, time to metamorphosis (development duration in days, between pupa and adult stage), time to emergence (development duration in days, between L1 and adult stage), adult survival (time from adult emergence to dead), and survivorship full span (using larvae that survived from L1 to dead adult stage). Sex ratio was estimated as the number of males relative to total emerged adults.

### Wing Length Measurements

To evaluate possible morphological variation in body size of adults reared with microorganism diets, we measured the size of males and females wings separately using standard methods of geometric morphometrics. In this study, we used the wings of adults emerged from larvae fed with standard diet Tetramin^®^, the yeasts *S. cerevisiae* and *Pseudozyma* sp., and the Gram-negative bacteria *E. coli* and *Asaia* sp. The wings (both sides) were removed from the thorax of individuals, mounted on Canadian balsam microscope glass slides and processed as reported by Lorenz et al. (2012). Images of the slides were digitized using a digital camera Leica DFC320 coupled to a Leica S6 (40×) stereoscope. To each image were registered coordinates *x* and *y* of 18 landmarks (Virginio et al., 2015) using TpsDig software V.2.05 (Rohlf, 2006). The wing size variations were assessed using measurements of the centroid size (CS) (Bookstein, 1992).

### Microorganism Staining and Larval Feeding Behavior

We decided to monitor the ingestion of live microorganisms labeled with fluorescein isothiocyanate (FITC) by *Ae. aegypti* larvae. The protocol of microorganism staining was performed according to Moraes et al. (2012). FITC-labeled microorganisms were resuspended in 3 mL of sterile water and added to 50 mL polypropylene tubes containing 7 mL of sterile water and 50 fourth instar larvae raised on Tetramin^®^. After 2 h incubation at 26°C, 10 larvae were dissected, and single guts were placed in microtubes with 100 μL of sterile NaCl 0.9% (w/v). Samples were homogenized by shaking the tube for 30 s at 25 Hz (MiniBeadBeater; Biospec Products, Bartlesville, OK, United States). The gut fluorescence detection was performed in a FlexStation 3 Multi-Mode Microplate Reader (Molecular Devices, San Jose, CA, United States) on λEx = 495 nm and λEm = 520 nm. Aliquots (10 μL) of microorganisms FITC-labeled were mounted on microscope glass slides for fluorescence observation in a Nikon Eclipse E200 (40×), fitted with a B-2A filter (Excitation Filter Wavelengths: 450–490; Dichromatic Mirror Cut-on Wavelength: 500; Barrier Filter Wavelengths: 515). Images were taken with a regular digital camera. Five experiments were performed.

### Protein and Carbohydrate Contents of Microorganism-Based Diets

Culture samples of 20 mL were centrifuged (7,500 × *g*, 20 min, 4°C). The supernatant was discarded, and cells were resuspended in 1 mL of water. Aliquots of 10 and 40 μL were withdrawn for protein and sugar measurements, respectively. We assessed total protein content using the bicinchoninic acid method procedure (Smith et al., 1985) and total carbohydrates were measured with the phenol-sulfuric method (Dubois et al., 1956). Eight experiments were performed for each diet.

### Measurement of the Energy Reserves in Single Individuals

Fourth instar newly molted larvae were individually weighed and immediately frozen for analysis of nutritional reserves. We quantified protein, total carbohydrate, glycogen, and lipids in single individual fourth instar entire larvae, gut and rest of the body for comparison. Larvae were reared as reported in item “Experimental Nutritional Protocol” and dissected as described in “Microorganism Staining and Larval Feeding Behavior.” The biochemical analysis was performed accordingly the Van Handel (1985a) method adapted by Foray et al. (2012). Protein content was measured as Bradford (1976) method using ovalbumin as a standard. Carbohydrates and glycogen were detected by an anthrone procedure using glucose as a standard (Van Handel, 1985a). Total lipid was determined in chloroform-methanol solvent solution by vanillin–phosphoric acid reaction (Van Handel, 1985b, 1988) using Glyceryl trioleate as standard (Van Handel and Lum, 1961). The assays were performed in 96-well microplates. Ten experiments were performed for each diet.

### Statistical Analysis

For all experiments, measurements were described using mean ± SEM. Developmental parameters (larval development, pupation, emergence, and survivorship) were analyzed using the GraphPad InStat v.3.01 (San Diego, CA, United States) and Excel^®^. The correlation among non-parametric variables was performed using the Log-rank (Mantel-Cox), Wilcoxon and Fisher tests. Tests for normality of the sample distribution were assessed by D’Agostino-Pearson omnibus test. Microorganism’s viability significance and measurement of protein and carbohydrate content on diets were examined with a *T*-test. Analysis of variance (ANOVA 1) was used in ingestion of live microorganisms labeled with FITC and measurement of the energy reserves in the whole larva. The wings morphometric statistical analyses were managed with the software MorphoJ (Klingenberg, 2011), and GraphPad InStat v.3.01 (San Diego, CA, United States). The normality and homoscedasticity of samples distribution were assessed by Shapiro–Wilks with the software Past3. In populations that had a Gaussian distribution, the parametric *T*-test was used based on means. In populations that did not, the non-parametric Mann–Whitney test was used based on medians.

## Results

**FIGURE 1:**
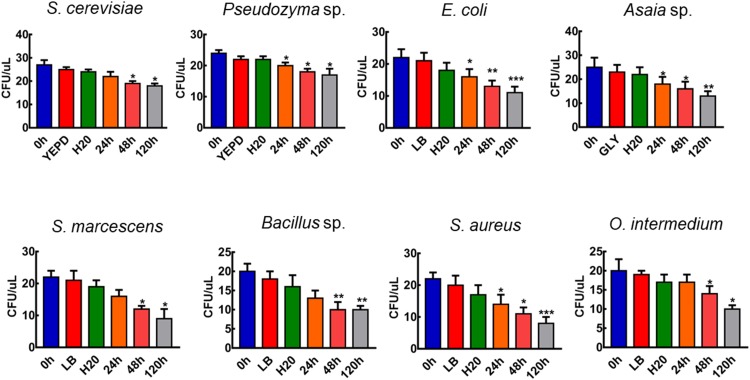
The viability of microorganisms in water. Total counts of CFU after centrifugation of microbial cells suspended in liquid media, water and after keeping the resuspended cells in water for 120 h. Figures are means ± SEM of 15 experiments each. (*T*-Test; ^∗^*p* > 0.05, ^∗∗^*p* < 0.01, ^∗∗∗^*p* < 0.005).

To verify if all the strains used in our experiments were viable on aquatic conditions we tested the viability of the microorganism cells in water. CFU counts revealed that re-suspension in water does not affect the viability and number of cells (*p* > 0.05, paired *T*-test, *n* = 15, Figure 1). *S. cerevisiae*, *S. marcescens*, *Bacillus* sp., and *O. intermedium* remained viable after being incubated in water for 48 h, and *Pseudozyma* sp., *E. coli*, *Asaia* sp., and *S. aureus* were viable until 24 h (Figure 1). These data demonstrate that it is possible to expose larvae to live cells and that these microbial cells might be used as a nutritional source.

### Estimates of Development and Survivorship for *Ae. aegypti* Reared Using Exclusive Microorganism-Based Diets

To assess the development and survivorship until the adult stage of *Ae. aegypti* reared exclusively with microorganism-based nourishment at immature stages, we tested four strains of Gram-negative bacteria were used: *S. marcescens* (SM365), *E. coli* (D31), *Asaia* sp. (A1), and *O. intermedium*; two strains of Gram-positive bacteria, *Bacillus* sp. and *S. aureus*; the yeasts: *S. cerevisiae* (S14) and *Pseudozyma* sp. (Pa1), a genus of microalga *Chlorella* sp., and a species of cyanobacteria (blue–green algae), *A. platensis* (*Spirulina*). The biological life attributes measured for the *Ae. aegypti* Rockefeller strain under controlled laboratory conditions are presented from Supplementary Tables S1–S6 and Tables 1–5, and summarized as follows.

**Table 1:** Average pupation time for females and males in days.

	Females ♀				Males ♂			
Diet	Pupae *n*	Mean ± *SEM*	Median	*p*-value	Pupae *n*	Mean ± *SEM*	Median	*p*-value
Tetramin^®^	88	5.4 ± 0.05	5	–	61	5.2 ± 0.05	5	–
*Pseudozyma* sp.	46	7.2 ± 0.22	7	*p* < 0.0001	83	6.1 ± 0.1	6	*p* < 0.0001
*S. cerevisiae*	62	8.4 ± 0.06	8	*p* < 0.0001	87	7.9 ± 0.07	8	*p* < 0.0001
*E. coli*	29	16.0 ± 0.8	15	*p* < 0.0001	58	15.6 ± 0.5	15	*p* < 0.0001
*S. marcescens*	0	NA	NA	NA	2	NA	NA	NA
*Bacillus* sp.	0	NA	NA	NA	2	NA	NA	NA
*Asaia* sp.	16	11.4 ± 0.6	10	*p* < 0.0001	25	9.9 ± 0.2	9	*p* < 0.0001
*S. aureus*	5	23 ± 3	22	*p* < 0.0001	11	22 ± 2	17	*p* < 0.0001
*O. intermedium*	1	NA	NA	NA	7	28 ± 3	28	*p* < 0.0001
*Chlorella* sp.	6	68 ± 4	69	*p* < 0.0001	21	59 ± 2	56	*p* < 0.0001
*A. platensis*	9	38 ± 1	39	*p* < 0.0001	19	34 ± 1	34	*p* < 0.0001

*NA, not available. Differences were analyzed with Log-rank and Wilcoxon.*

The developmental time from L1 to pupae differed significantly (*p* < 0.0001) between the diets. Larvae of the standard group fed with Tetramin^®^ developed in 5.3 ± 0.04 days. *Pseudozyma* sp. and *S. cerevisiae* (mean 6.5 ± 0.12; 8.1 ± 0.05 days) developed faster than the larvae on other microorganism-based diets. Larvae fed with the *Chlorella* sp. take longer to develop until pupation with a mean time of 61.5 ± 2.09 days until pupa (Supplementary Table S1). *S. marcescens* (42.0 ± 3.0 days) and *Bacillus* sp. (34.0 ± 0.0 days) showed the longest time to achieve pupal stage compared with the other bacterial diets.

The next biological parameter evaluated was the duration of the metamorphosis period of larvae in adult mosquitoes. No significant differences (*p* > 0,05) were detected in diets that used *Asaia* sp. (2.0 ± 0.04 days; *p* = 0.6804), *O. intermedium* (2.0 ± 0.0 days; *p* = 0.3652), *Chlorella* sp. (1.9 ± 0.07 days; *p* = 0.0978) and *A. platensis* (1.9 ± 0.09; *p* = 0.1042) compared to the standard group fed with Tetramin^®^ (2.09 ± 0.02 days) (Supplementary Table S2). Time from L1 to adult emergence differed significantly (*p* < 0.0001) among each diet. Time until the adult stage was higher to *Chlorella* sp. (Supplementary Table S3).

The survival of adults maintained only with water was evaluated once a day until confirmation of the death of all insects. The diets containing *E. coli* (6.2 ± 0.3 days) and *Asaia* sp. (5.5 ± 7.52 days) showed the closest survival rates compared to the standard diet (8.9 ± 0.1 days). *Bacillus* sp. (2.0 ± 0.0 days), *S. aureus* (2.3 ± 6.1 days), and *O. intermedium* (2.5 ± 2.1 days) revealed the lowest survival rates (Supplementary Table S4). The full lifespan from L1 to adult death is significantly (*p* < 0.0001) different between diets (Supplementary Table S5).

Biological parameters were analyzed separately by gender to disclose possible sex-specific effects in development rates and survivorship. The development time of L1 to pupae differed significantly in females and males (*p* < 0.001; Table 1) from different diets. Larvae fed with yeast diet developed faster in both genders (mean 7.2 ± 0.2; 8.4 ± 0.06 days for females; 6.1 ± 0.1; 7.9 ± 0.07 days for males) than larvae on bacteria and microalgae diets. Male development time until pupa is shortest than female larvae in all diets used (Table 1). No significant differences (*p* = 0.7867) were detected for female adult metamorphosis on diets containing *Asaia* sp. However, a significant (*p* < 0.0001) effect was observed among all the other diets compared to the standard group fed with Tetramin^®^ (Table 2).

**Table 2:** Average metamorphosis time for females and males in days.

	Females ♀				Males ♂			
Diet	Adults *n*	Mean ± *SEM*	Median	*p*-value	Adult *n*	Mean ± *SEM*	Median	*p*-value
Tetramin^®^	88	2.1 ± 0.03	2	–	61	2.1 ± 0.04	2	–
*Pseudozyma* sp.	46	3.0 ± 0.09	3	*p* < 0.0001	83	2.4 ± 0.05	2	*p* < 0.0001
*S. cerevisiae*	62	2.5 ± 0.06	2.5	*p* < 0.0001	87	2.5 ± 0.05	3	*p* < 0.0001
*E. coli*	29	2.4 ± 0.09	2	0.0002	58	2.2 ± 0.05	2	0.0865
*S. marcescens*	0	NA	NA	NA	2	NA	NA	NA
*Bacillus* sp.	0	NA	NA	NA	2	NA	NA	NA
*Asaia* sp.	16	2.1 ± 0.09	2	0.7867	25	2.0 ± 0.04	2	0.4904
*S. aureus*	5	2.6 ± 0.2	3	0.0013	11	2.4 ± 0.2	2	0.0098
*O. intermedium*	1	NA	NA	NA	7	2 ± 0	2	0.4347
*Chlorella* sp.	6	2 ± 0	2	*p* < 0.0001	21	2 ± 0	2	0.1784
*A. platensis*	9	2 ± 0	2	*p* < 0.0001	19	2 ± 0	2	0.2003

*NA: Not available. Differences were analyzed with Log-rank and Wilcoxon.*

Male development time until adult metamorphosis differed significantly (*p* < 0.0001) solely on yeasts diets, and no significant differences were observed among the other diets (Table 3). Female average development time until adult emergence was longest than males. In both genders a significant difference (*p* < 0.0001) was detected when compared microorganism-based diets with the group fed with the standard diet Tetramin (Table 3). The survival of adults (males or females) differs significantly (*p* < 0.0001) between diets. The average survival time of each female adult varied from 2.6 ± 0.93 to 5.1 ± 0.73 days across the different dietary supply. Females fed with *A. platensis* (5.1 ± 0.73 days), *Asaia* sp. (4.8 ± 0.39 days) and *E. coli* (4.6 ± 0.40 days) exhibited an elongated average survival (Table 4).

**Table 3:** Average emergence time for females and males in days.

	Females ♀				Males ♂			
Diet	Adults *n*	Mean ± *SEM*	Median	*p*-value	Adults *n*	Mean ± *SEM*	Median	*p*-value
Tetramin^®^	88	7.5 ± 0.07	7	–	61	7.3 ± 0.08	7	–
*Pseudozyma* sp.	46	9.7 ± 0.22	9	*p* < 0.0001	83	8.5 ± 0.1	8	*p* < 0.0001
*S. cerevisiae*	62	10.9 ± 0.06	11	*p* < 0.0001	87	10.4 ± 0.07	10	*p* < 0.0001
*E. coli*	29	18.4 ± 0.8	18	*p* < 0.0001	58	17.8 ± 0.5	17	*p* < 0.0001
*S. marcescens*	0	NA	NA	NA	2	NA	NA	NA
*Bacillus* sp.	0	NA	NA	NA	2	NA	NA	NA
*Asaia* sp.	16	13.6 ± 0.4	13	*p* < 0.0001	25	12.0 ± 0.2	11	*p* < 0.0001
*S. aureus*	5	25 ± 2	24	*p* < 0.0001	11	23 ± 2	20	*p* < 0.0001
*O. intermedium*	1	NA	NA	NA	7	30 ± 3	30	*p* < 0.0001
*Chlorella* sp.	6	69 ± 4	70	*p* < 0.0001	21	61 ± 2	58	*p* < 0.0001
*A. platensis*	9	40 ± 1	41	*p* < 0.0001	19	35 ± 1	36	*p* < 0.0001

*NA, not available. Differences were analyzed with Log-rank and Wilcoxon.*

**Table 4:** Adult average survival time in females and males.

	Females ♀				Males ♂			
Diet	Adults *n*	Mean ± *SEM*	Median	*p*-value	Adults *n*	Mean ± *SEM*	Median	*p*-value
Tetramin^®^	88	8.4 ± 0.1	8	–	61	9.5 ± 0.1	10	–
*Pseudozyma* sp.	46	3.3 ± 0.1	3	*p* < 0.0001	83	5.6 ± 0.1	6	*p* < 0.0001
*S. cerevisiae*	62	3.6 ± 0.1	4	*p* < 0.0001	87	5.5 ± 0.2	4	*p* < 0.0001
*E. coli*	29	4.6 ± 0.4	4	*p* < 0.0001	58	7.0 ± 0.4	7	*p* < 0.0001
*S. marcescens*	0	NA	NA	NA	2	NA	NA	NA
*Bacillus* sp.	0	NA	NA	NA	2	NA	NA	NA
*Asaia* sp.	16	4.8 ± 0.3	4	*p* < 0.0001	25	6.1 ± 0.2	7	*p* < 0.0001
*S. aureus*	5	2.6 ± 0.9	2	*p* < 0.0001	11	2.1 ± 0.3	2	*p* < 0.0001
*O. intermedium*	1	NA	NA	NA	7	2.7 ± 0.6	2	*p* < 0.0001
*Chlorella* sp.	6	3.7 ± 0.2	4	*p* < 0.0001	21	3.5 ± 0.1	3	*p* < 0.0001
*A. platensis*	9	5.1 ± 0.7	4	*p* < 0.0001	19	3.8 ± 0.2	4	*p* < 0.0001

*NA, not available. Differences were analyzed with Log-rank and Wilcoxon.*

**FIGURE 2:**
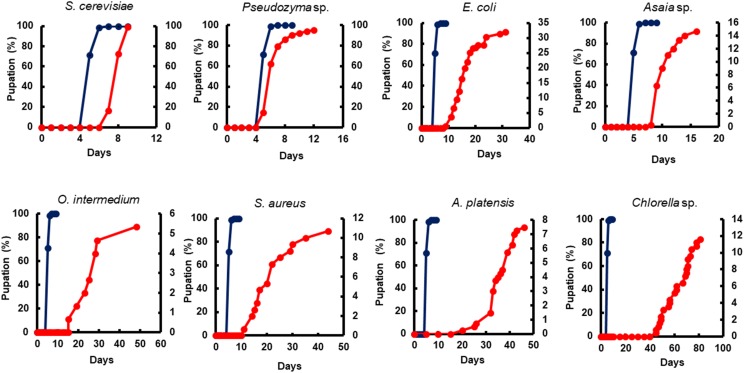
The impact of microorganism-based diets on pupation time. A representative pupation curve is comparing larvae fed with Tetramin (Dark blue line, left *y*-axis) and larvae fed with microbial cells (red line, right *y*-axis). The dietary supply was administered only at L1. Dead individuals were removed daily.

**FIGURE 3:**
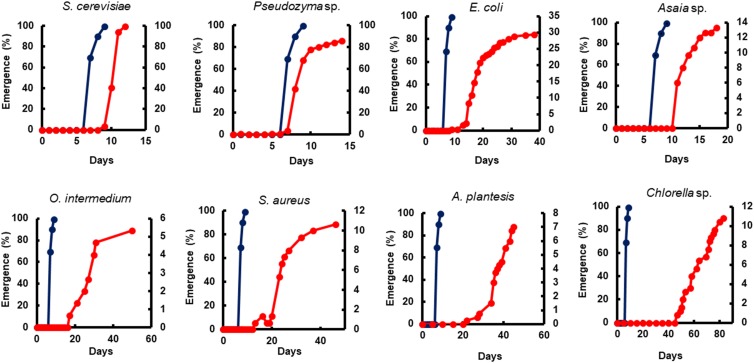
The impact of microorganism-based diets on adult emergence. A representative emergence curve is comparing larvae fed with Tetramin (Dark blue line, left *y*-axis) and larvae fed with microbial cells (red line, right *y*-axis). The dietary supply was administered only at L1. Dead individuals were removed daily.

**FIGURE 4:**
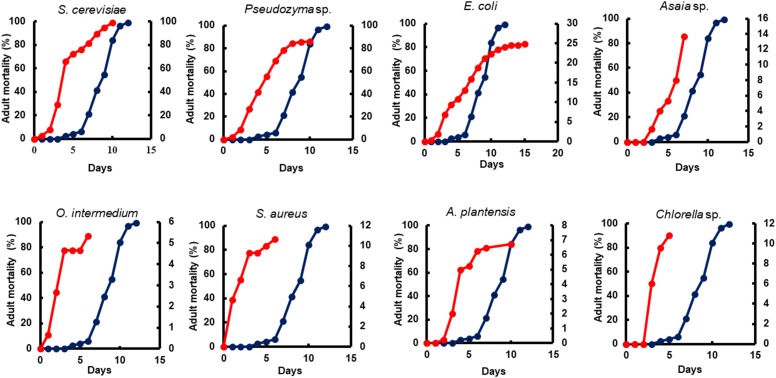
The impact of microorganism-based diet on adult survival. A representative survival curve comparing larvae fed with Tetramin (Dark blue line, left *y*-axis) and larvae fed with microbial cells (red line. Right *y*-axis). The dietary supply was administered only at L1. Survival was assessed daily.

**FIGURE 5:**
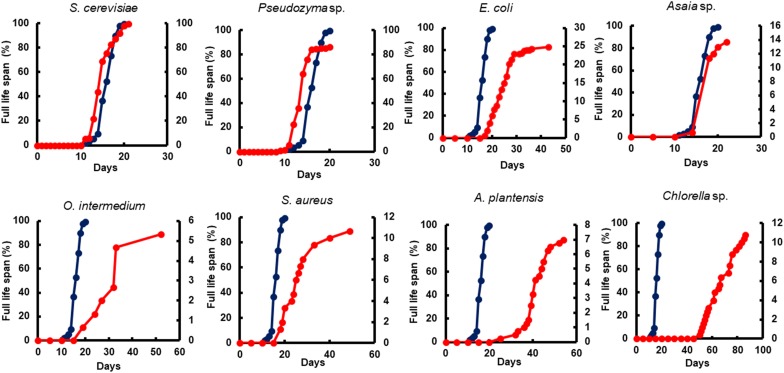
The impact of microorganism-based diets on total life span. A representative life span curve is comparing larvae fed with Tetramin (dark blue line, left *y*-axis) and larvae fed with microbial cells (red line, right *y*-axis). The dietary supply was administered only at L1. Survival was assessed daily.

The adult survival pattern observed in males differed partially from females. The average observed in the survival span for males varied from 2.1 ± 0.39 to 7.0 ± 0.44 days. Males fed with *E. coli* (7.0 ± 0.44) and *Asaia* sp. (6.1 ± 0.24 days) displayed the same longest survival observed for females. Additionally, males fed with *Pseudozyma* sp. (5.6 ± 0.19 days) and *S. cerevisiae* (5.5 ± 0.28 days) also presented a long survival (Table 4). The full lifespan of males and females from L1 larvae to pupation differ significantly among diets (*p* < 0.0001). The average life span varied from 13.0 ± 0.23 to 73.5 ± 4.74 days for females and 14.0 ± 0.17 to 65.0 ± 2.32 days for males (Table 5). The overall adult sex ratio was more male-biased (Supplementary Table S6). The development time of L1 to pupae, adult emergence, survivorship, and lifespan of all dietary studied have been depicted graphically in Figure 2–5.

**Table 5:** The full lifespan of females and males in days.

	Females ♀				Males ♂			
Diet	Adults *n*	Mean ± *SEM*	Median	*p*-value	Adults *n*	Mean ± *SEM*	Median	*p*-value
Tetramin^®^	88	15.9 ± 0.1	16	–	61	16.8 ± 0.2	17	–
*Pseudozyma* sp.	46	13.0 ± 0.2	13	*p* < 0.0001	83	14.0 ± 0.1	14	*p* < 0.0001
*S. cerevisiae*	62	14.0 ± 0.1	14	*p* < 0.0001	87	15.9 ± 0.2	15	*p* < 0.0001
*E. coli*	29	23.0 ± 0.8	21	*p* < 0.0001	58	24.8 ± 0.6	25	*p* < 0.0001
*S. marcescens*	0	NA	NA	NA	2	NA	NA	NA
*Bacillus* sp.	0	NA	NA	NA	2	NA	NA	NA
*Asaia* sp.	16	18.4 ± 0.5	18	*p* < 0.0001	25	18.1 ± 0.08	18	*p* < 0.0001
*S. aureus*	5	28 ± 2	26	*p* < 0.0001	11	26 ± 2	26	*p* < 0.0001
*O. intermedium*	1	24 ± 0	24	NA	7	33 ± 3	33	*p* < 0.0001
*Chlorella* sp.	6	73 ± 4	75	*p* < 0.0001	21	65 ± 2	62	*p* < 0.0001
*A. platensis*	9	43 ± 1	45	*p* < 0.0001	19	39 ± 1	40	*p* < 0.0001

*NA, not available. Differences were analyzed with Log-rank and Wilcoxon.*

Yeast diets revealed a similar development time to standard diet Tetramin^®^. The diets of bacteria and microalgae, in the opposite, presented a lethargic larval development (Figure 2, 3). Regarding adult mortality, *E. coli* and *Asaia* sp. presented the highest survival mean in days, surpassing even the yeast diets (Figure 4). The full lifespan was extended in immature stages fed with bacteria and microalgae diets. These results suggest a possible badly nourishment which could breed smaller larvae with difficulty to attain the critical mass that is necessary for metamorphosis (Figure 5; Telang et al., 2007). All the compiled biological development data were detailed in the Supplementary Spreadsheet S1.

**FIGURE 6:**
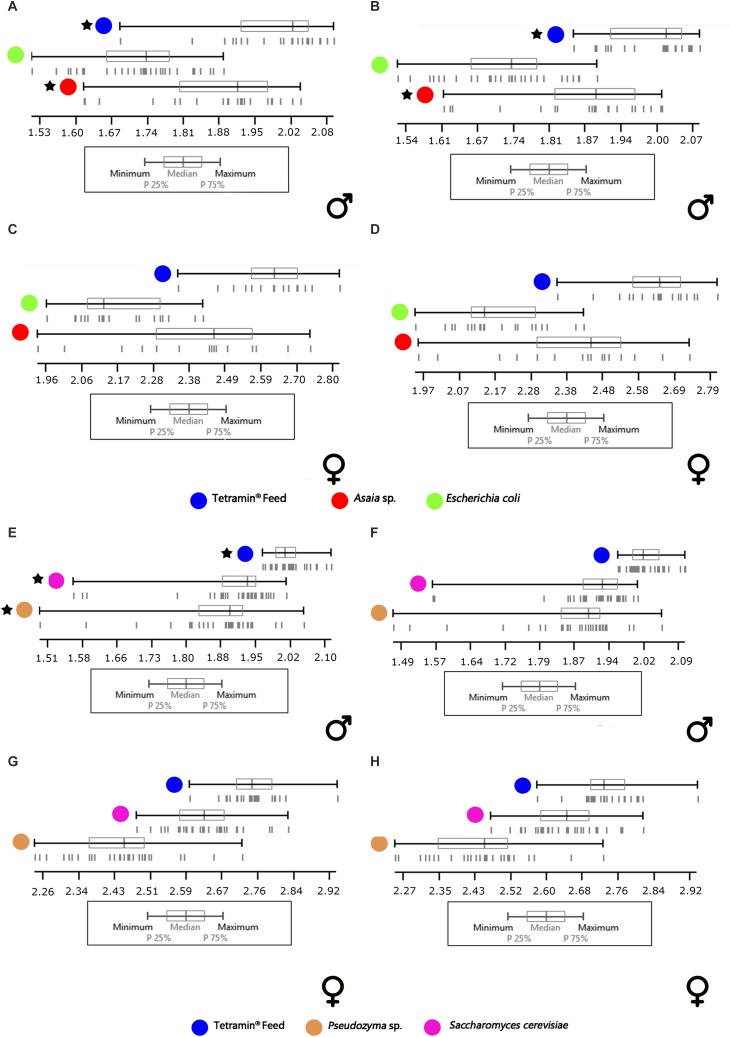
Descriptive statistics of right **(B,D,F,H)** and left **(A,C,E,G)** wing centroid sizes (in mm) of males and females from different diets. **(A–D)** Comparison between control diet (Tetramin^®^) and Gram-negative bacteria (*Asaia* sp. and *E. coli*). **(E–H)** Comparison between control diet [Tetramin (R)] and Yeasts (*Pseudozyma* sp. and *S. cerevisiae*). Vertical lines: individuals. Asterisks: Non-normal distribution.

Concerning the wing centroid size analysis, which is herein used as a predictor of body size, the majority of samples showed a normal distribution (*p* > 0.05, Supplementary Tables S7, S8). In general terms, the comparisons between microorganism-based diets and the standard group (with normal and non-normal distribution, respectively), showed a significant difference of sizes in both genders and wing sides (*p* < 0.05). The isometric size of males and females reared in Tetramin^®^ showed larger adults than all the other diets (Figure 6). On average, among the experimental diets (excluding the standard group), the largest females were reared in *S. cerevisiae*, followed by *Pseudozyma* sp., *Asaia* sp., and *E. coli* diet. The males were largest in *S. cerevisiae*, *Asaia* sp., *Pseudozyma* sp., and *E. coli* diet, respectively (Figure 6).

### Nutritional Evaluation of Diets, Energetic Reserve Accumulation Measurement and Feeding Behavior of *Ae. aegypti* Larvae Reared Exclusively With Microorganisms

Nutritional quality influences the physiology of *Ae. aegypti* immature stages directly. To evaluate the impact of quality of two important macronutrients on larval breeding, we quantified the protein and carbohydrate contents present in the dietary supplies used. Differences in the nutritional composition of the microorganism diets were detected and may have influenced the immature stages development time (Table 6). *S. cerevisiae* and *Pseudozyma* sp. diets have higher amounts of protein (116 ± 6.1 mg; 75 ± 6.9 mg) and carbohydrate (180 ± 9.2 mg; 98 ± 3.8 mg) than the bacterial diets (7.0 ± 0.6 mg to 15.4 ± 1.4 mg and 0.3 ± 0.1 mg to 1.6 ± 0.02 mg). Yeast diets, on the other hand, have less protein than *A. platensis* (341 ± 34 mg) and *Chlorella* sp. (300 ± 17.5 mg) diets. Conversely, the content of carbohydrates was higher on diets with yeasts (180 ± 9.2 mg; 98 ± 3.8 mg) compared to the diets based in *A. platensis* (35 ± 4 mg) and *Chlorella* sp. (26 ± 1.5 mg).

Four major energetic components (protein, carbohydrate, glycogen, and lipids) and the body weight were measured in individual fourth instar entire larvae and compared with the contents recovered from their guts and rest of body tissues. The results are summarized in Table 7. The absolute values of the nutritional reserves differed significantly (*p* < 0.0001) among individuals compared with the standard group Tetramin^®^. Larvae fed with *S. cerevisiae, Pseudozyma* sp, *E. coli*, and *Asaia* sp. accumulate the highest amounts of protein (194 ± 18 mg to 89 ± 3 mg), carbohydrates (103 ± 1.3 mg to 16 ± 0.3 mg), glycogen (12 ± 0.8 to 3 ± 0.4 mg) and lipids (71 ± 4.9 to 27 ± 0.8 mg). The nutritional reserve accumulation in *A. platensis*, *Chlorella* sp., *S. aureus*, *S. marcescens*, *Bacillus* sp., and *O. intermedium* showed the lowest amounts of energetic components (protein, carbohydrate, glycogen, and lipid) and these results are in agreement with the longer developmental rates observed in the experiments above (Supplementary Tables S1–S5 and Tables 1–5). Mean body weight differed significantly with larval diet (Table 7). Only *S. cerevisiae* and *Pseudozyma* sp. did not differ significantly compared with the standard diet.

**FIGURE 7:**
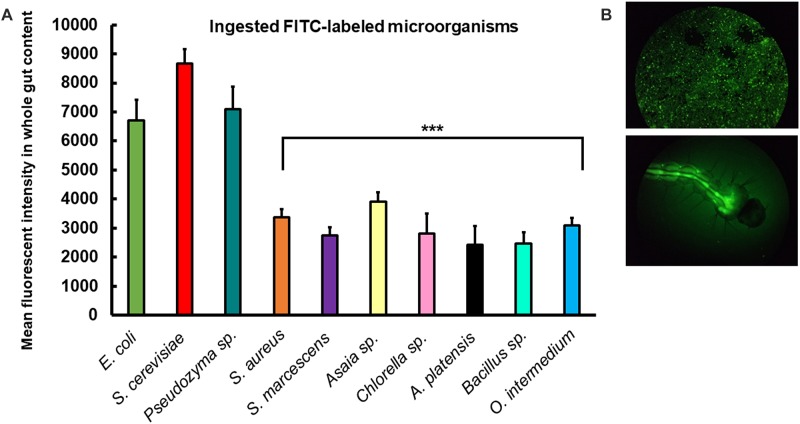
*Ae. aegypti* larvae consume different microorganisms offered in diets. **(A)** Mean fluorescent intensity in the intestine of larvae 2 h after placement of FITC-labeled microorganisms into rearing water. Columns present mean values with 95% confidence intervals for each diet (ANOVA 1; ^∗^*p* < 0.05; ^∗∗∗^*p* < 0.001). Figures are means ± SEM of 5 experiments with ten larvae each. **(B)** Epifluorescent images of microscope slide glass with FITC-labeled *E. coli* (D31) and individual larva 2 h after placing FITC-labeled *E.coli* (D31) into the rearing water.

To further investigate the rates of ingestion of the different microorganisms by larvae, the consumption rate of each diet was measured by fluorescence in the gut of individual larvae after 2 h of incubation with FITC-labeled cells. Larvae fed with *S. cerevisiae*, *Pseudozyma* sp., and *E. coli* consumed more food than larvae fed with the other diets after 2 h (Figure 7). Our results showed that *Ae. aegypti* larvae actively consumed all the microorganisms used, but with some preference for the microorganisms above (Supplementary Figures S1, S2).

## Discussion

The nutrition environment experienced by larvae strongly influences the physiology and behavior of mosquitoes. The current work evaluated the impact of nourishment from live microbes in the development and survival of *Ae. aegypti*. The organisms selected for feeding were strains of Gram-negative bacteria *S. marcescens* (SM365), *E. coli* (D31), *Asaia* sp. (A1), and *O. intermedium* (Om17), Gram-positive bacteria *Bacillus* sp. and *S. aureus*, yeast-like fungi *S. cerevisiae* (S14), and *Pseudozyma* sp. (Pa1), the cyanobacteria (blue–green algae) *A. platensis* (also known as *Spirulina*), and the marine microalga *Chlorella* sp. All microorganisms used in this paper showed stability in water until 120 h. Therefore, we decided to perform the feeding experiments by dispersing the strains directly in rearing water under sterile conditions.

### General Developmental Parameters in *A. aegypti* Raised on Microorganisms-Based Diets

All diets showed important differences in developmental and survival rates when compared individually to the standard group Tetramin^®^. Larva fed with yeast take less time to achieve pupation than all the other microorganism-based diets tested (Figure 2). Parameters as adult emergence (Figure 3), survival (Figure 4) and full lifespan (Figure 5) showed a slight delay in developmental rates when compared to the standard group Tetramin^®^. Due to the satisfactory developmental rates in all life parameters tested, and the rapid growth speed of cultures in low-cost media, *S. cerevisiae*, and *Pseudozyma* sp. seem to be suitable candidates for diets in the mass rearing of mosquitoes or the regular laboratory breeding (Imam et al., 2014). Trager (1935a,b) studies pioneered larval nutrition, demonstrating that *Ae. aegypti* larvae can reach adulthood being fed only with yeast powder. Confirming the results obtained by Trager, 1935a showed that autoclaved yeasts suspended in CaCl_2_ 0.01% is sufficient for the full larval lifespan of *Ae. aegypti*. Souza et al. (2016) conducted a study based on feeding larvae with a specific diet containing live or dead *S. cerevisiae* cells only. The data obtained in this paper corroborated the works described previously, that highlighted the ability of *Ae. aegypti* larvae to feed and digest living yeast cells through the enzyme beta-1,3-glucanase. Yeast and fungi can also be suitability as a paratransgenic vehicles or integrated pest management (IPM) tools.

**Table 6:** Protein and carbohydrate contents in microorganism-based diets.

Diets	Proteins (mg)	Carbohydrates (mg)
Tetramin^®^	212 ± 3	207 ± 2
*S. cerevisiae*	116 ± 6^∗∗∗∗^	180 ± 9^∗∗∗∗^
*Pseudozyma* sp.	75 ± 6^∗∗∗∗^	98 ± 3^∗∗∗∗^
*E. coli*	15 ± 1^∗∗∗∗^	1,6 ± 0,02^∗∗∗∗^
*S. aureus*	7,4 ± 0,4^∗∗∗∗^	1,0 ± 0,1^∗∗∗∗^
*S. marcescens*	7,0 ± 0,6^∗∗∗∗^	0,5 ± 0,05^∗∗∗∗^
*Bacillus* sp.	6,7 ± 0,8^∗∗∗∗^	0,3 ± 0,1^∗∗∗∗^
*Asaia* sp.	10,3 ± 1,6^∗∗∗∗^	1,4 ± 0,04^∗∗∗∗^
*O. intermedium*	7,7 ± 0,6^∗∗∗∗^	1,0 ± 0,04^∗∗∗∗^
*Chlorella* sp.	300 ± 10^∗∗∗∗^	26 ± 1^∗∗∗∗^
*A. platensis*	340 ± 30^∗∗∗∗^	35 ± 4^∗∗∗∗^

*(ANOVA 1; ^∗∗∗∗^p < 0.001).*

**Table 7:** Nutritional reserve amounts of proteins, soluble carbohydrates, glycogen and total lipids in *A. aegypti* larvae raised in different microorganism-based diets, and their tissues.

	Tetramin^®^	*S. cerevisiae*	*Pseudozyma* sp.	*A. platensis*	*Chlorella* sp.	*E. coli*	*S. aureus*	*S. marcescens*	*Bacillus* sp.	*Asaia* sp.	*O. intermedium*
**Protein (mg)**											
Larvae	250 ± 30	190 ± 10^∗^	171 ± 6^∗∗^	60 ± 7^∗∗∗∗^	50 ± 10^∗∗∗^	117 ± 6^∗∗∗∗^	64 ± 0.7^∗∗∗∗^	48 ± 1^∗∗∗∗^	52 ± 0.8^∗∗∗∗^	89 ± 3^∗∗∗∗^	55 ± 0.7^∗∗∗∗^
Gut	77 ± 1	44 ± 2^∗∗∗^	48 ± 1^∗∗∗^	4 ± 0.4^∗∗∗∗^	6 ± 0.4^∗∗∗∗^	14 ± 2^∗∗∗∗^	9 ± 0.3^∗∗∗∗^	6 ± 0.3^∗∗∗∗^	3 ± 0.5^∗∗∗∗^	10 ± 1^∗∗∗∗^	4 ± 0.2^∗∗∗∗^
Carcass	169 ± 2	133 ± 5	116 ± 9^∗∗^	50 ± 10^∗∗∗∗^	43 ± 4^∗^	89 ± 5^∗∗∗∗^	50 ± 2^∗∗∗∗^	39 ± 1^∗∗∗∗^	46 ± 1^∗∗∗∗^	76 ± 3^∗∗∗∗^	51 ± 2^∗∗∗∗^
**Carbohydrate (mg)**											
Larvae	125 ± 3	103 ± 1	82 ± 0.8	10 ± 0.3^∗∗∗∗^	9.2 ± 0.4^∗∗∗∗^	21 ± 3^∗∗∗∗^	13 ± 1^∗∗∗∗^	5 ± 0.3^∗∗∗∗^	5 ± 0.4^∗∗∗∗^	16 ± 0.3^∗∗∗∗^	11 ± 0.1^∗∗∗∗^
Gut	23 ± 2	17 ± 0.8^∗∗∗^	16 ± 0.6^∗∗∗∗^	2 ± 0.1^∗∗∗∗^	2 ± 0.1^∗∗∗∗^	1.4 ± 0.1^∗∗∗∗^	3 ± 0.1^∗∗∗∗^	0.7 ± 0.1^∗∗∗∗^	0.8 ± 0.1^∗∗∗∗^	1 ± 0.1^∗∗∗∗^	2 ± 0.1^∗∗∗∗^
Carcass	82 ± 3	77 ± 1^∗∗^	61 ± 0.4^∗∗∗^	6.9 ± 0.3^∗∗∗∗^	6.7 ± 0.3^∗∗∗∗^	13 ± 0.6^∗∗∗∗^	7 ± 0.4^∗∗∗∗^	4 ± 0.2^∗∗∗∗^	4 ± 0.2^∗∗∗∗^	14 ± 0.4^∗∗∗∗^	8 ± 0.1^∗∗∗∗^
**Glycogen (mg)**											
Larvae	20 ± 0.5	12 ± 0.8^∗∗∗^	10 ± 0.3^∗∗∗^	2 ± 0.1^∗∗∗∗^	3 ± 0.2^∗∗∗∗^	4 ± 0.9^∗∗∗∗^	3 ± 0.4^∗∗∗∗^	1 ± 0.5^∗∗∗∗^	1 ± 0.3^∗∗∗∗^	3 ± 0.4^∗∗∗∗^	2 ± 0.1^∗∗∗∗^
Gut	5 ± 0.2	3 ± 0.5	3 ± 0.5	0.8 ± 0.05^∗^	0,9 ± 0.1^∗^	0.3 ± 0.04^∗^	0.3 ± 0.1^∗^	0.2 ± 0.1^∗^	0.1 ± 0.04^∗∗^	0.2 ± 0.02^∗^	0.3 ± 0.03^∗∗^
Carcass	11 ± 0.5	6 ± 0.3^∗∗^	6 ± 0.5^∗∗^	1 ± 0.1^∗∗^	2 ± 0.2^∗∗^	3 ± 0.3^∗∗∗^	3 ± 0.2^∗∗∗^	1 ± 0.1^∗∗∗^	1 ± 0.1^∗∗∗^	2 ± 0.3^∗∗∗^	1 ± 0.1^∗∗∗^
**Lipid (mg)**											
Larvae	111 ± 1	71 ± 4^∗^	79 ± 5^∗∗^	19 ± 1^∗∗^	16 ± 0.7^∗∗∗^	36 ± 4^∗^	23 ± 4^∗∗∗∗^	7 ± 0.4^∗∗∗∗^	6 ± 0.6^∗∗∗∗^	27 ± 0.8^∗∗^	17 ± 0.4^∗∗∗∗^
Gut	32 ± 3	17 ± 1^∗∗^	22 ± 0.6^∗∗^	3 ± 0.5^∗∗∗∗^	3 ± 0.4^∗∗∗∗^	1 ± 0.4^∗∗∗∗^	1 ± 0.1^∗∗∗∗^	0.2 ± 0.04^∗∗∗∗^	0.3 ± 0.03^∗∗∗∗^	2 ± 1^∗∗∗∗^	1 ± 0.1^∗∗∗∗^
Carcass	70 ± 4	53 ± 1^∗∗^	51 ± 2^∗∗^	14 ± 1^∗∗∗^	12 ± 0.9^∗∗∗^	28 ± 0.2^∗∗∗^	17 ± 2^∗∗∗^	6 ± 0.2^∗∗∗∗^	5 ± 0.2 ^∗∗∗∗^	22 ± 0.7^∗∗∗^	15 ± 0.8^∗∗∗^
**Fresh Weight (mg)**											
Larvae	7 ± 0.0004	6 ± 0.001	6 ± 0.0004	3 ± 0.0003^∗^	2 ± 0.0003^∗^	3 ± 0.0003^∗^	3 ± 0.0003^∗^	3 ± 0.001^∗^	3 ± 0.001^∗∗^	3 ± 0.0003^∗^	3 ± 0.0003^∗∗^
Gut	3 ± 0.001	3 ± 0.0002	3 ± 0.0003	1 ± 0.0003 ^∗∗^	1 ± 0.0003^∗∗^	1 ± 0.0002^∗∗^	1 ± 0.0002^∗∗^	1 ± 0.0001^∗∗∗^	1 ± 0.0002^∗∗∗^	1 ± 0.0001^∗∗^	1 ± 0.0003^∗∗^
Carcass	5 ± 0.002	4 ± 0.0001	4 ± 0.0003	2 ± 0.001^∗^	2 ± 0.0003^∗^	2 ± 0.0003^∗∗∗^	2 ± 0.0003^∗∗∗^	2 ± 0.001^∗∗∗^	3 ± 0.001^∗∗∗^	2 ± 0.0003^∗^	2 ± 0.0001^∗∗^

*^∗^p < 0.05, ^∗∗^p < 0.01, ^∗∗∗^p < 0.005, ^∗∗∗∗^p < 0.001.*

Interestingly, the symbiont *Wickerhamomyces anomalus (Saccharomycetales)* can be found in the gut and reproductive organs of some mosquito vector species. This symbiont can be easily cultured in cell-free media and seem to be a good candidate for the expression of effector molecules in the gut of mosquito vectors (Ricci et al., 2011b). Murphy et al. (2016) demonstrated that genetically modified *S. cerevisiae* could be used as biopesticide through oral delivery of species-specific dsRNA. This application as biopesticide decreases larval survivorship, reduces locomotor activity and reproductive fitness in the insect pest *Drosophila suzukii*. This yeast biopesticide approach could be adapted to a large number of species once many interactions have been observed between sylvatic yeasts and insect species as Diptera, Coleoptera, and Hymenoptera (Gonzalez, 2014; Abrieux and Chiu, 2016). The authors also postulate that biopesticide design may be favored in the managing of an insect pest that both consumes yeast as food and has systemic RNAi.

Studies of biological control agents based on entomopathogenic fungi have been reported in several vector mosquitoes. The potential of *Metarhizium anisopliae* fungus was tested in *Anopheles gambiae*, *Ae. aegypti*, *Aedes albopictus*, *Culex quinquefasciatus* (Alves et al., 2002; Scholte et al., 2007; de Paula et al., 2008; Pereira et al., 2009; Fang et al., 2011). Genetic-engineered *M. anisopliae* inhibited *Plasmodium* sp. development within the mosquito and prevented malaria infection in *Anopheles* (Fang *et al.*, 2011). In *Ae. aegypti*, *A. albopictus*, and *C. quinquefasciatus*, the full lifespan of *M. anisopliae*-contaminated mosquitoes was significantly reduced and showed high mortality rates compared to uninfected mosquitoes (Alves et al., 2002; Scholte et al., 2007; de Paula et al., 2008; Pereira et al., 2009). Recently, two strains of *M. anisopliae* were tested against *Ae. aegypti* and besides the increase in mortality, the fungus also reduced egg laying (Jemberie et al., 2018). The fungi: *Lagenidium giganteum* and *Leptolegnia chapmanii* were also tested as promising biological control agents for use against *Ae. aegypti* adults (McCray et al., 1973; McInnis and Zattau, 1982). The development of innovative strategies using yeast has, therefore, potential as an eco-productive alternative for the management of mosquito-borne diseases.

Bacteria are considered the most common microorganism present in the nourishment of mosquito larvae (Laird, 1956, 1988; Christophers, 1960). Previous studies reported that bacteria could be used as a unique food requisite to mosquito growth (Hinman, 1932; Rozeboom, 1935). Larvae fed with *S. marcescens*, *Bacillus* sp, *S. aureus*, *O. intermedium*, *S. aureus*, *E. coli*, and *Asaia* sp. showed a severe delay to achieve pupal stage when compared to the standard group Tetramin^®^ (Supplementary Table S1). Other biological parameters as metamorphosis (Supplementary Table S2), emergence (Figure 3), survival (Figure 4) and full lifespan also strongly affected (Figure 5). Studies obtained by Dickson et al. (2017) provide the concept that larval exposure to different bacterial communities during larval development can drive variation in *Ae. aegypti* adult traits. Therefore, the results observed here are similar to other studies reported. Noteworthy, *E. coli* and *Asaia* sp. take result in shorter developmental times into pupa than the other bacterial diets (Figure 2). The survival span for larvae fed with *E. coli* and *Asaia* sp. were superior to those observed in diets using *S. cerevisiae* and *Pseudozyma* sp. *E. coli* and *Asaia* sp. seem to be the best bacterial models for laboratory-reared larvae up until now.

Food stress was expected to reduce survival, however, larva fed with *Asaia* sp. and *E. coli* had higher adult survival (Supplementary Table S4). There are different ways to interpret these results. Nutritional stress can increase the life-span by a hormetic model (Mattson, 2008). Larvae fed with *E. coli* and *Asaia* sp. might be presenting a stress-induced response, hormesis, that can be an overcompensation to environmental, nutritional stress (Calabrese, 2001). Hormesis induces cellular protective mechanisms through an increased in gene expression, working as a key regulator of many cellular defenses that allow survival in response to stress (Lin et al., 2000; Motta et al., 2004; Heilbronn et al., 2005). Enhanced levels of heat shock proteins (HSPs) and antioxidants to cellular maintenance are also considered as part of the hormetic mechanism (Gems and Partridge, 2008; Mattson, 2008). The beneficial effects of hormesis on survival and longevity have been described for years, and our results might thus exemplify a beneficial carry-over effect of the hormetic stress on larval development, reinforcing the *Ae. aegypti* phenotypic plasticity in limiting environments (Aznar et al., 2018). Previous studies had already shown that larvae reared in restricted diets might be associated with prolonged life in *Ae. aegypti* (Joy et al., 2010; Zeller and Koella, 2016) and *Anopheles* sp. (Vantaux et al., 2016).

The positive correlations between the presence of microbiota and larval development might be another way to explain the results observed in larvae fed with *Asaia* sp. and *E. coli*. The acetic acid bacterium genus *Asaia* have been shown to be stably associated with larvae and adults of anophelines and *Ae. aegypti* (Favia et al., 2007; Damiani et al., 2010; Ricci et al., 2012a; Rossi et al., 2015). Mitraka et al. (2013) showed that a diet supplemented with *Asaia* in the *Anopheles gambiae* larval environment had a significant boost in developmental rate and Chouaia et al. (2012) observed a delayed larval development in *Anopheles* mosquitoes deprived of *Asaia* bacterial symbionts. Besides the effects on development rates, a possible mutual exclusion or a competition between *Asaia* and *Wolbachia* may contribute to explain the inability of *Wolbachia* to colonize the female reproductive organs of anophelines, inhibiting its vertical transmission and explaining the absence of *Wolbachia* infection in *Ae. aegypti* and in the majority of natural populations of *Anopheles* mosquitoes (Rossi et al., 2015). These results drive us to believe that *Asaia* may play a significant role in mosquito larval development. Although the molecular nature of the developmental improvement caused by the *Asaia* symbiont needs to be identified, these bacteria can be considerate as candidate paratransgenic vehicle for the control of mosquito-borne diseases (Favia et al., 2008; Ricci et al., 2012b; Mitraka et al., 2013).

Coon et al. (2017) showed that each mosquito species including *Ae. aegypti* contains a simple bacterial community and that the composition of bacterial gut communities can also be strongly influenced by diet. Their results also showed that axenic larvae could not develop, but several community members and *E. coli* can rescue the larval development. Using *E. coli* K-12 as a model for studies of molecular interactions that underlie bacteria-dependent growth of larvae into adults, Coon et al. (2017) unveiled one of the molecular mechanisms involved in mosquito development. They showed that bacteria through the cytochrome b oxidase gene mediate a reduction of oxygen levels in the digestive tract of larvae, working as a signal for ecdysone-induced molting. Thereby, *E. coli* plays an essential role in mosquito development and may have important implications to be used in symbiont-based control techniques for disabling the growth of larvae into mosquito adults.

*S. marcescens*, *Bacillus* sp., *O. intermedium*, and *S. aureus* had a lower adult rate survival contrary to results described in diets with *Asaia* sp. and *E. coli* (Supplementary Table S4). The effect of a restricted diet in mosquito larva is a rather speculative issue. Previous studies reported that dietary restrictions can lead to longer development rates (Olivo et al., 1979; Tun-Lin et al., 2000; Arrivillaga and Barrera, 2004; Couret et al., 2014; Levi et al., 2014; Aznar et al., 2018), with larvae extending time to achieve the pupal stage (Chambers and Klowden, 1990; Telang et al., 2007; Foster et al., 2012; Banerjee et al., 2015). Some individuals with slower growth rates try to counterbalance this deficit through compensatory growth (Wilson and Osbourn, 1960). This ecological factor allows that once the same nutritionally deprived individuals have the opportunity to acquire more food, speed up or slow down the growth rates to employ the food resources in the maintenance of important biologicals traits as reproduction and survival (Dmitriew, 2011; Dmitriew and Rowe, 2011; Zeller and Koella, 2016).

*Bacillus* sp. and *S. marcescens* presented only two adults (Supplementary Table S1). *S. marcescens* is recognized by its entomopathogenic properties in some conditions (Flyg et al., 1980; Flyg and Xanthopoulos, 1983). However, in this study, it does not appear to be associated with infections in *Ae. aegypti* larvae. In the present study, larvae fed with bacterial diets, including *S. marcescens*, remained in the third instar for several weeks. Other studies have been shown that withstand to starvation could be measured by the time spent in the third larval instar without adequate nourishment (Wigglesworth, 1942). The duration of development from the L1 larval stage to adult mosquito is faster when food is abundant (Tun-Lin et al., 2000); thus, the prolongation in third instar larvae observed in our results may indicate a state of malnutrition. We did not observe the presence of melanized-killed larvae or any specific phenotype more related to pathogenicity. However, previous studies have already reported that food restriction alters immunological traits in *Ae. aegypti* (Grimstad and Walker, 1991; Alto et al., 2005, 2008; Muturi et al., 2011; Telang et al., 2012). More studies are necessary to evaluate the influence that the diets used in this study exert on the immune system of *Ae. aegypti* larvae.

Lowering of growth rates may be a response to dietary stress and adaptive behavior in calorie-depleted environments (Arendt, 1997; Badyaev, 2005). It is possible that *Ae. aegypti* larvae raised under bacterial diets suffer nutritional depletion, resulting in developmental delays. In natural environments, mosquitoes are commonly found with reduced sizes and low energy reserves. This remarkable capacity of withstanding starvation situations and recovery later in more favorable conditions can justify the great success for the establishment of these insects in the environment (Barrera, 1996; Barrera and Medialdea, 1996; Zeller and Koella, 2016). Therefore, our data show that *Ae. aegypti* larva can develop in all bacterial diets tested, even at a higher ecological cost.

In natural conditions, the biomass of algae is the major content of mosquito larvae guts (Hinman, 1930; Garros et al., 2008). Thus, algae seem to play an important role in the development and survival of larvae. *Ae. aegypti* larvae were not able to develop in microalgae diets with the standard concentration established for the other diets (16 mg/larva), so we adjusted the values offered to lower concentrations (3 mg/larvae). The development failure observed in the standard concentration (data not shown) might have been caused by several factors among them the junction between a controlled photoperiod environment as well as a low larval density. Together these factors might have accelerated the exponential microalgae growth triggering a toxicological response by larvae which impaired their development.

Larvae fed with the microalgae *Chlorella* sp or with *A. platensis* (3 mg/larvae) showed a delay in development similar to the observed in bacterial diets, but with shorter adult survivals (Supplementary Table S4). Kivuyo et al. (2014) evaluated larval survival and the development rate of the aquatic stage using green filamentous algae and dry powdered filamentous algae as diet. The survivorship and pupation rates in algae food diet had the worst performance between all foods assessed. Some species of algae seem to be resistant to digestion and are discarded entirely after passage through the larval gut (Laird, 1988). However, there is no evidence that *Chlorella* sp. and *A. platensis* belong to this specific group.

Interestingly, a genus of *Chlorella* was used as a larvicide against *Ae. aegypti* by Borovsky et al. (2016). *Chlorella desiccata* was engineered to express an insect peptide hormone, the trypsin modulating oostatic factor (TMOF), that is recognized by *Ae. aegypti* ovaries and controls the translation of the gut’s trypsin mRNA. Feeding mosquito larvae with transformed *C. desiccata* cells kill by starvation 60% of *Ae. aegypti* larvae in 4 days (Borovsky et al., 2016). *A. platensis* (*Spirulina*) have never been used in nourishment experiments with mosquito larvae before. Our results showed that *Chlorella* and *A. platensis* could be used as a food source by mosquito larvae, but with slower developmental rates. Their rapid grow under heterotrophic culture, with a relatively low cost, make these microalgae interesting candidates for future methods of vector control through genetic engineering (Dawson et al., 1997; Madkour et al., 2012).

### Sex-Related Parameters in *A. aegypti* Raised on Microorganism-Based Diets

The biological parameters evaluated in *Ae. aegypti* were analyzed separately for males and females in all diets (Tables 1–5 and Supplementary Table S6). The results showed that, as already described in the literature, males take less time to reach adulthood and survive longer. When mosquitoes are fed only water as adults, the only nutritional resources available lie in reserve acquired during immature stages. The transference of reserves from the larval nutrition to the mosquito adult stage using only microbial cells as nourishment has never been investigated before. Thus, it is possible that males show more withstand to starvation during the larval stage and a lower nutritional threshold for pupation than females. These traits might have influenced the better developmental rates observed in males and increased their survival. The measured lifespan of adult males and females fed with nectar is approximately 9 weeks and around 12–17 weeks for females that were also blood fed (Putnam and Shannon, 1934). We were not able to find available data in the literature to compare the mortality rates of males and females under laboratory conditions without any nutritional stimulus. Aznar et al. (2018) reported that treatments with scarcity or excess of food might preferentially influence the proportions and survival of females over males. They have shown that females exhibit a larger extension of development time in response to food deprivation than males, and relate this result to a male fitness advantage, ability of an individual to survive, reproduce and spread genes, this advantage is obtained when males emerge early and can copulate with non-mated females. The longer development time on females is similar to the observed under a competitive environment (Bedhomme et al., 2003). Under variated conditions, it is expected that females take more time to develop and spend more time to enhance their fitness abilities, achieving larger body sizes and thus boost their fecundity (Bedhomme et al., 2003; Wormington and Juliano, 2014). Previous field studies already reported a faster development in a male over female mosquito larvae, that showed a slower development and a higher mortality rate (Yates, 1979).

Imbalances in sex ratio (males: females) were observed in all diets tested (Supplementary Table S6). This male-biased sex ratio may result from underfeeding. Some studies support a sex-related difference in larval nutrient metabolism, possibly due to the earlier ecdysteroid peak in *Ae. aegypti* male during pupal-adult development (Brust, 1967; Whisenston et al., 1989; Chambers and Klowden, 1990; Puggioli et al., 2013; Balestrino et al., 2014). In field studies, distortion in sex ratio is frequent and associated to the slow development of female larvae and differential response of the sexes to egg hatching stimuli (Yates, 1979; Shroyer and Craig, 1981; Sims and Munstermann, 1983; Frank et al., 1985; Lounibos and Escher, 2008). Other studies observed a density-dependent alteration and a sex-specific response to a critical day period time, trough feedback mechanisms that are dependent on density or mortality by selective sexual predation (Chambers, 1985; Frank et al., 1985; Alto et al., 2012). The highest proportion of males observed in this study seem favorable to future studies using the Sterile Insect Technique (SIT). A greater male pupae production is important to SIT mass rearing and determine the number of males that can be selected for release in the natural environment (Puggioli et al., 2016).

In this study, it was also possible to realize that microbial diets (yeast and Gram-negative bacteria) influenced the wing size of *Ae. aegypti* adults, and presumably the whole body size, of both genders. The correlation of wing size with food concentration is interesting, mainly because the “centroid size” (Bookstein, 1992) is considered a more informative estimator of body size than the traditional size measurements (Jirakanjanakit and Dujardin, 2005; Jirakanjanakit et al., 2007). As previously mentioned, we quantified the wing centroid size as a read-out of the adult size. In general, the wings of the mosquitoes fed with Tetramin^®^ in their immature stages were larger than those that were fed with microorganism-based diets. Also, in all comparisons, females showed larger wings than males. Sexual dimorphism is present in wing shape and size of *Ae. aegypti* (Virginio et al., 2015; Sánchez and Liria, 2017). However, independently of the inherent wing sexual dimorphism, and some asymmetry, we recorded some patterns of differentiation. In males, the group fed with *E. coli* showed lower sizes than the *Asaia* sp. group, which in turn was more similar to Tetramin^®^. In females, *Asaia* sp. and *E. coli* were lower than Tetramin^®^, although *Asaia* sp. has been closer to the Tetramin^®^ diet. On the yeast diets, males of *Pseudozyma* sp. and *S. cerevisiae* showed similar scores, and females of the *Pseudozyma* sp. were lower than *S. cerevisiae*, both being lower than the Tetramin^®^ score. Among all the microorganism-based diets tested, *S. cerevisiae* showed the wing sizes that are closest to the Tetramin^®^ diet. Jong et al. (2017), showed significant shorter wings in *A. albopictus* under sub-optimal food availability. Jirakanjanakit et al. (2007) and Aznar et al. (2018) reported that low food concentration in *Ae. aegypti* immature stages could alter mosquito size. Those results support this study, suggesting a strong influence of microbial food composition on mosquito body size.

### Nutritional Analysis of Microorganism-Based Diets and Nutrient Acquisition by Larvae

The differences reported in the developmental time and adult size with the microbial diets are consistent with other studies on environment stress (Badyaev, 2005). Response to stress conditions such as nutritional limitation (Bubliy et al., 2000), extreme temperatures (Sisodia and Singh, 2009) and larval crowding (Imasheva and Bubliy, 2003) in *Drosophila* have been studied and related to genetic modifications. Schneider et al. (2011) demonstrated a genotype variation in *Ae. aegypti* adult size in response to larval food depletion, reporting wide phenotypic plasticity and adaptive behavior to changing environments. Aznar et al. (2018), suggested that the variety in food stress conditions in the natural habitat can increase genetic modifications in *Ae. aegypti*. This genetic variety, as mutation and recombination rates, is usually hidden under regular food conditions and facilitates the development of novel adaptations to adverse environments (Badyaev, 2005).

The next step was to analyze the nutritional quality through the measurement of the main energetic components of diets. Our goal was to observe if these developmental and size variations are a result of malnourishment. The nutritional requirements of mosquitoes are divided into two majority classes: macronutrients (energetic nutrients) and micronutrients (non-energetic nutrients) (Singh and Brown, 1957; Foster, 1995; Arrese and Soulages, 2010; Canavoso et al., 2011; Rivera-Pérez et al., 2017). Aquatic environments with abundant nutritional richness supply all the energy necessary to mosquito larvae metamorphosis. Carbohydrates and proteins are among the main nutritional requirements of *Ae. aegypti* (Singh and Brown, 1957).

The nutritional values of the diets were evaluated through protein and total sugar quantification (Table 6). *Chlorella* sp and *A. platensis* showed higher protein amounts than the other diets, including the standard diet Tetramin^®^. These results were already expected once both species are rich in proteins and can be employed even for human consumption (Mühling, 2000; Guccione et al., 2014; Bleakley and Hayes, 2017). Yeast diets also presented robust protein values. *S. cerevisiae* has more protein than *Pseudozyma* sp. Souza et al. (2016) showed that *S. cerevisiae* contains higher protein amounts than the standard diet Cat food. However, Tetramin^®^ seems to be more nutritive and present a superior protein amount. *Bacillus* sp., *S. marcescens*, *S. aureus*, and *O. intermedium* showed lower protein values than *E. coli* and *Asaia* sp. These results coincide with the developmental pattern observed (Supplementary Tables S1–S5). As previously mentioned, protein and amino acids consumption are directly related to growth and development in mosquitoes (Golberg and De Meillon, 1948; Merritt et al., 1992). Scarce consumption of proteins during the immature stages might interfere in life cycle duration, adult emergence, body size and fecundity (Singh and Brown, 1957; Dadd, 1977). Thus, our results are in line with other studies, showing that badly nourishment leads to delayed larval development and adults with low energetic reserves (Timmermann and Briegel, 1999; Arrivillaga and Barrera, 2004; Telang et al., 2007; Banerjee et al., 2015; Zeller and Koella, 2016).

The sugar amounts in *S. cerevisiae* and *Pseudozyma* sp. were higher than in the other diets. *S. cerevisiae* did not show significant differences (*p* < 0,005) when compared to the standard diet (Table 6). All the bacterial diets had low sugar contents and *E. coli* and *Asaia* sp. presented the best values, reinforcing our hypothesis that both bacteria are better sources for larval feeding. *Chlorella* sp. and *A. platensis* presented high sugar values. However, in contrast to the protein amounts, their sugar content is lower than the yeast or the standard diet. Carbohydrate is directly associated with pupation (Chambers and Klowden, 1990; Telang et al., 2007). Carbohydrate storage is associated to pupal commitment and larval growth (Sneller and Dadd, 1977; Van Handel, 1988; Telang et al., 2007), and the low levels observed in our results suggest that larvae have not sufficient sugar to achieve the pupal stage in these diets, resulting in developmental delay. Although the microalgae species had a significant amount of protein and sugar, the development time in these diets was prolonged, leading us to believe that *Ae. aegypti* larvae may not assimilate nutrients adequately from these organisms.

Besides the nutritional quality measurements, we analyzed the nutrient reserves of *Ae. aegypti* larvae. We have undertaken a comparative study in the larval development rates and their storage of protein, free carbohydrates, glycogen and lipids concerning different dietary conditions. There is a positive correlation between body mass and caloric reserves stored during the larval stage, triggering endocrine responses that lead to insect molting (Chambers and Klowden, 1990). Insects must achieve a minimum weight in the immature stage in order for continuing their development (Nijhout and Williams, 1974; Nijhout, 1975; Lounibos, 1979; Safranek and Williams, 1984; Chambers and Klowden, 1990; Davidowitz et al., 2003; Lan and Grier, 2004; Mirth et al., 2005). In *Ae. aegypti* larvae, the metamorphic capacity depends on nutritional reserves and needs a minimum critical mass that is estimated between 2.7 and 3.2 mg (Telang et al., 2007). Critical mass is defined as the mass that results in 50% of starved larvae achieving the pupal stage. That usually occurs 24 h after the transformation into the final fourth instar, in optimal conditions (Chambers and Klowden, 1990; Davidowitz et al., 2003; Lan and Grier, 2004; Telang et al., 2007). In this minimum period these larvae require to acquire food so that at least 50% of them pupate and emerge as adults, and at this age, the ecdysteroid production begins to rise (Telang et al., 2007). Nutrient intake and energetic accumulation are important factors to metamorphosis. Most larvae that are starved after reaching their critical weight will molt, but if larvae are starved before they have achieved the critical weight, metamorphosis is delayed, or they eventually die without initiating this process (Chambers and Klowden, 1990; Davidowitz et al., 2003; Telang and Wells, 2004).

Insects fed exclusively with bacteria and microalgae seem unable to accumulate sufficient nutrients during the larval stage to reach the minimum critical mass required for pupation in regular time (Table 7). Restrictive food amount in *Ae. aegypti* larvae delay pupation, but larvae remain able to pupate even in underfeeding conditions if sufficient energetic accumulation has been done in previous larval stages (Telang et al., 2007). Thus, the delay observed in our results in the metamorphosis rates might afford to larvae an additional period to feed, grow, and meet the critical mass to achieve the pupal stage. In all diets tested, the energetic larval contents were lower than in larvae fed with the standard diet Tetramin^®^. These results were expected, once the insect physiological parameters were strongly affected (Supplementary Tables S1–S5). Additional evidence for developmental delays comes from studies in *Manduca sexta*. The underfeeding in *M. sexta* during the last larval stage resulted in death or a delay in metamorphosis (Nijhout and Williams, 1974).

The protein storage in larvae fed with bacteria or algae showed the lowest proteins levels and *Asaia* sp. and *E. coli* showed better levels than the others including *Chlorella* sp. (54 ± 19.1 mg) and *A. platensis*. Although microalgae have a high protein content, *Ae. aegypti* seems not able to store this protein satisfactorily. The energetic reserves in gut and rest of body of all diets tested were proportional to values obtained in whole larvae, and the rest of the body showed higher contents than the gut (Table 7). Yeast-fed larvae showed the highest levels of protein, suggesting that *Ae. aegypti* larvae can process and store this protein efficiently. In immature stages, proteins play an important role in metabolic processes as well for adults of several insect species (Hagen et al., 1984; Zucoloto, 1988). In immature stages ingestion of proteinaceous food is essential for growth, survival, nutritional reserve for the pupation and utilization in the adult stage, principally for egg production (Chan et al., 1990). Our results are in line with the decline in body size observed in *Ae. aegypti*, *Culex pipiens*, *Anopheles albimanus*, and *Anopheles gambiae* caused by different feeding regimes (Timmermann and Briegel, 1999).

Carbohydrates content in bacterial and microalgae diets showed significant storage differences (*p* < 0.0001). Larvae fed with *S. cerevisiae* and *Pseudozyma* sp. presented carbohydrates reserves similar to the standard diet, showing no significant differences (*p* > 0.05). As expected, *E. coli* and *Asaia* sp. had better carbohydrates storage amounts than other bacteria and microalgae, reinforcing the potential of these diets. Carbohydrate is necessary for optimal growth and developmental rates in larvae, and nutritional environment with low levels of sugar leads to a substantially delayed growth (Sneller and Dadd, 1977). Previous studies with *M. sexta* suggested the dependence of sugar ingestion to reaching the pupa stage and the requirement to both dietary sugar and protein to growth until the adults (MacWhinnie et al., 2005). Carbohydrate role in *Ae. aegypti* larvae development is associated with pupal commitment and an inverse relation between hemolymph trehalose levels, and juvenile hormone titers have been described (Jones et al., 1981; Chambers and Klowden, 1990; Telang et al., 2007). Therefore, our data confirm that sugar and other digestible carbohydrates are required for adult stage maintenance and larval development.

The larvae glycogen storage showed significant differences (*p* = 0.0001) in all diets tested. Bacterial diets resulted in low glycogen contents, and *Asaia* sp. and *E. coli* showed higher rates than other bacteria diets. Larvae fed with *S. cerevisiae* and *Pseudozyma* sp. showed better glycogen levels, confirming the excellent storage capacity of *Ae. aegypti* in yeast diets. Past studies reported a critical threshold of larval glycogen as a stimulant to metamorphosis through a drastic drop in juvenile hormone titer and a concomitant increase in ecdysone that trigger molting (Chambers and Klowden, 1990; Timmermann and Briegel, 1999). Telang et al. (2007) reported that the timing of ecdysteroid release is not critical to start the larval-pupal molt for *Ae. aegypti* larvae, however, both the ecdysteroid titer and the nutritional condition of fourth instars are determinant factors in initiating the metamorphic molt. Earlier studies indicated that larval nutrient reserves (protein, lipid, and glycogen) are important for egg production and the endocrine regulation of egg development in *Ae. aegypti* and *Ochlerotatus atropalpus* (Telang et al., 2006). High levels of glycogen and protein overtake a threshold set in the insect nervous system that activates ovarian ecdysteroid production and inhibits juvenile hormone biosynthesis by the *corpora allata*, which together enable vitellogenesis and egg production. Without sufficient threshold levels, the *corpora allata* increase the juvenile hormone levels secretion, decreasing ovarian ecdysteroid production and as consequence egg maturation is delayed (Telang et al., 2006).

The low glycogen reserve in *Ae. aegypti* larvae fed with bacteria and microalgae might have affected the insect neuroendocrine system, resulting in the delaying of pupation time, due to a lack of the minimum glycogen threshold required for a successful molt (Nijhout and Wheeler, 1982; Chambers and Klowden, 1990; Lan and Grier, 2004; Noriega, 2004; Telang and Wells, 2004; Margam et al., 2006; Telang et al., 2006, 2007). Glycogen storage seems to be a larval strategy valid to withstand starvation when the larva is waiting for additional nutrition during the last larval instar, under badly nourishment conditions (Timmermann and Briegel, 1999). This strategy might be used by larvae fed with *E. coli* and *Asaia* sp., which showed higher glycogen levels and prolonged their survival.

Levels of lipids were lowest in larvae fed with *S. marcescens* and *Bacillus* sp. These diets showed the worst developmental rates (Supplementary Table S1) with a severe delay in all biological parameters evaluated (Supplementary Tables S1–S5). Although, the other experimental diets have also shown significant and substantial differences (*p* < 0.05) in the lipid contents. Lipid reserve influence pupal commitment, and the endocrine regulation of egg development in autogenous and anautogenous female mosquitoes (Briegel, 1990b; Foster, 1995; Ziegler and Ibrahim, 2001; Briegel et al., 2002; Zhou et al., 2004; Telang et al., 2006). Under favorable nutritional conditions, lipids start accumulating after glycogen has reached a plateau (Van Handel, 1984). Environments with nutritional stress lead to drastic reductions in whole-body lipid, protein and carbohydrate contents (Briegel, 1990b). Due to the diets poor in nutrients (Table 6), *Ae. aegypti* larvae storage lesser amounts of energetic components (Table 7), affecting the developmental rates directly. The metamorphic capacity clearly depends on all four nutrient reserves in different ways.

Nutrients accumulated by larvae are correlated with adult emergence and body size (Telang et al., 2007; Zeller and Koella, 2016). Nutrition, temperature and larval density also influence growth, development, energy reserves, egg production, longevity of adult females, immunity, vector capacity and insecticide-resistance (Stearns and Koella, 1986; Briegel, 1990a,b; Kitthawee et al., 1992; Lyimo et al., 1992; Juliano and Stoffregen, 1994; Ameneshewa and Service, 1996; Telang et al., 2006, 2007; Murrell and Juliano, 2008; Reiskind and Lounibos, 2009; Arrese and Soulages, 2010; Muturi et al., 2011; Kulma et al., 2013; Murray et al., 2013). Larvae fed with *S. cerevisiae* and *Pseudozyma* sp. were not affected in body weight, what is expected as these diets showed good developmental rates, high nutritional quality, and efficient energetic accumulation. The fresh mass of larva fed with bacteria and microalgae were significantly lower (*p* < 0.05) when compared to controls. In natural environments, it is common to find mosquitoes with reduced sizes and low energy reserves (Zeller and Koella, 2016). Telang et al. (2007) showed that nutritional stress leads to smaller adults with reduced hemocyte numbers. Muturi et al. (2011) reported that nutritional stress during larval development cause changes in phenotype and immunity of mosquitoes and increased susceptibility of these adults to pathogens. Therefore, the nutritional quality of larvae in diets with microorganisms may affect not only the entire life history of *Ae. aegypti*, but also their size, immunity, and vector competence.

### Larval Preference for Ingestion of Microorganisms

Our results showed that *Ae. aegypti* larvae consumed all microorganisms used at different rates (Figure 7). The guts of larvae fed with *S. cerevisiae*, *Pseudozyma* sp., and *E. coli* presented high mean fluorescent intensity after 2 h of feeding. The lower consumption rates in other diets might suggest a possible food preference. Despite that, more studies must be performed, increasing the feeding exposure time. We have not found studies involving food preference in *Ae. aegypti* larvae, hence exploring this subject is necessary for a better understanding of larval physiology.

## Conclusion

Our results provide new knowledge into the effect of microorganism-based diets in different larval biological parameters as developmental rates, pupation time, emergence, survival, lifespan, and wing size. Larvae fed with bacteria and microalgae shown lethargic development and low survival, due to bad nourishment and low energetic reserve accumulation. *Asaia* sp. and *E. coli* seem the best bacterial models for future studies aiming the development of symbiont-based methods for mosquito control. Larvae fed with yeasts showed developmental rates that are similar to the standard diet Tetramin^®^, being nutritional rich and providing high energetic storage. *S. cerevisiae* and *Pseudozyma* sp. seem suitable candidates to improve mosquito laboratory breeding and a low-cost diet to mosquito mass rearing. *Ae. aegypti* larvae ingested all the tested microorganisms 2 h after addition of them to the water. Therefore, *Ae. aegypti* larvae showed very high plasticity about feeding, being able to develop under different microbial diets.

## Author Contributions

RS and FV performed the experiments and analyzed the data. RS, FV, TR, LS, JB, and FG conceived the study. All authors wrote and revised the manuscript.

## Conflict of Interest Statement

The authors declare that the research was conducted in the absence of any commercial or financial relationships that could be construed as a potential conflict of interest.
