# Supplementary material for: Microorganism-Based Larval Diets Affect Mosquito Development, Size and Nutritional Reserves in the Yellow Fever Mosquito Aedes aegypti (Diptera: Culicidae)
Source: Front Physiol. 2019 Apr 9;10:152. doi: 10.3389/fphys.2019.00152 (PMC6465640; doi:10.3389/fphys.2019.00152)
Supplement: Supplementary file 1 [file Presentation_1.pdf]

Supplementary Material

Spreadsheet 1. Biological life parameters of *Aedes aegypti* raised on microorganism-based diets (Spreadsheet complete). Excel file.

Supplementary Table 1. Average pupation time in days of *Aedes aegypti* larvae reared with microorganisms. Differences were analyzed with Log-rank and Wilcoxon.

Diet	n_i	Pupae (n)	Mean ± SEM	Median	Pupation (%)	p value
Tetramin®	150	150	5.3 ± 0.04	5	100	-
Pseudozyma sp	150	143	6.5 ± 0.1	6	95	$p < 0.0001$
Saccharomyces cerevisiae	150	150	8.1 ± 0.05	8	100	$p < 0.0001$
Escherichia coli	300	114	16.7 ± 0.5	15	38	$p < 0.0001$
Serratia marcescens	400	4	42 ± 3	42	1	NA
Bacillus sp	150	2	34.0 ± 0.0	34	1	NA
Asaia sp	300	44	10.5 ± 0.3	10	15	$p < 0.0001$
Staphylococcus aureus	150	33	24 ± 1	22	22	$p < 0.0001$
Ochrobactrum intermedium	150	9	28 ± 3	28	6	$p < 0.0001$
Chlorella sp	250	28	61 ± 2	61	11	$p < 0.0001$
Arthrospira platensis	400	30	35 ± 1	35	8	$p < 0.0001$

n_i: Number of individuals used

NA: Not available.

Supplementary Table 2. Average metamorphosis time in days of *Aedes aegypti* reared with microorganisms in larval stages. Differences were analyzed with Log-rank and Wilcoxon.

Diet	Adults (*n*)	Mean ± SEM	Median	Metamorphosis(%)	*p* value
Tetramin®	149	2.1 ± 0.02	2	99	-
Pseudozyma sp	129	2.4 ± 0.05	2	90	$p < 0.0001$
Saccharomyces cerevisiae	149	2.5 ± 0.04	3	99	$p < 0.0001$
Escherichia coli	87	2.3 ± 0.1	2	76	*p* 0.0003
Serratia marcescens	2	2.5 ± 0.5	3	50	NA
Bacillus sp	2	2.0 ± 0.0	2	100	NA
Asaia sp	41	2.0 ± 0.04	2	93	*p* 0.6804
Staphylococcus aureus	16	2.4 ± 2.0	2	48	$p < 0.0001$
Ochrobactrum intermedium	8	2.0 ± 0.0	2	89	*p* 0.3652
Chlorella sp	27	1.9 ± 0.07	2	96	*p* 0.0978
Arthrospira platensis	28	1.9 ± 0.09	2	93	*p* 0.1042

NA: Not available.

Supplementary Table 3. Average emergence time in days of *Aedes aegypti* reared with microorganisms in larval stages. Differences were analyzed with Log-rank and Wilcoxon.

Diet	Adults (*n*)	Mean ± SEM	Median	Emergence (%)	*p* value
Tetramin®	149	7.4 ± 0.05	7	99	-
Pseudozyma sp	129	8.9 ± 0.1	9	86	$p < 0.0001$
Saccharomyces cerevisiae	149	10.6 ± 0.05	11	99	$p < 0.0001$
Escherichia coli	87	18.0 ± 0.4	17	29	$p < 0.0001$
Serratia marcescens	1	44 ± 4	45	0	NA
Bacillus sp	2	36.0 ± 0.0	36	1	NA
Asaia sp	41	12.6 ± 0.3	12	14	$p < 0.0001$
Staphylococcus aureus	16	25 ± 2	24	11	$p < 0.0001$
Ochrobactrum intermedium	8	28 ± 3	29	5	$p < 0.0001$
Chlorella sp	27	63 ± 2	63	11	$p < 0.0001$
Arthrospira platensis	28	37 ± 1	37	7	$p < 0.0001$

NA: Not available.

Supplementary Table 4. Average mean survival in days of *Aedes aegypti* reared with microorganisms in larval stage. Differences were analyzed with Log-rank and Wilcoxon.

Diet	Adults (*n*)	Mean ± SEM	Median	*p* value
Tetramin®	149	8.9 ± 0.1	9	-
Pseudozyma sp	129	4.7 ± 0.1	5	$p < 0.0001$
Saccharomyces cerevisiae	149	4.8 ± 0.1	4	$p < 0.0001$
Escherichia coli	87	6.2 ± 0.3	6	$p < 0.0001$
Serratia marcescens	2	3.0 ± 0.0	3	NA
Bacillus sp	2	2.0 ± 0.0	2	NA
Asaia sp	41	5.6 ± 0.2	6	$p < 0.0001$
Staphylococcus aureus	16	2.3 ± 0.3	2	$p < 0.0001$
Ochrobactrum intermedium	8	2.5± 0.2	3	$p < 0.0001$
Chlorella sp	27	3.4± 0.1	3	$p < 0.0001$
Arthrospira platensis	28	4.0± 0.3	4	$p < 0.0001$

NA: Not available.

Supplementary Table 5. Full lifespan in days of *Aedes aegypti* reared with microorganisms in larval stage. Differences were analyzed with Log-rank and Wilcoxon.

Diet	Adults (*n*)	Mean ± SEM	Median	*p* value
Tetramin®	149	16.3 ± 0.1	16	-
Pseudozyma sp	129	13.7 ± 0.1	14	$p < 0.0001$
Saccharomyces cerevisiae	149	15.1 ± 0.1	15	$p <0.0001$
Escherichia coli	87	24.2 ± 0.5	24	$p < 0.0001$
Serratia marcescens	2	46.5 ± 0.0	47	NA
Bacillus sp	2	38.0 ± 0.0	38	NA
Asaia sp	41	18.2 ± 0.2	18	$p < 0.0001$
Staphylococcus aureus	16	26 ± 2	25	$p < 0.0001$
Ochrobactrum intermedium	8	31 ± 3	33	$p < 0.0001$
Chlorella sp	27	64 ± 3	66	$p < 0.0001$
Arthrospira platensis	28	39 ± 1	41	$p < 0.0001$

NA: Not available.

Supplementary Table 6. Sex ratio of emerged adults. Differences were analyzed with Fisher test.

Diet	Males n ♂	Females *n*♀	Sex Ratio	*p* value
Tetramin®	61	88	69	-
Pseudozyma sp	83	46	180	0.0001
Saccharomyces cerevisiae	87	62	140	0.0037
Escherichia coli	58	29	200	0.0002
Serratia marcescens	2	0	NA	NA
Bacillus sp	2	0	NA	NA
Asaia sp	25	16	156	0.0328
Staphylococcus aureus	11	5	220	0.0604
Ochrobactrum intermedium	7	1	700	0.0215
Chlorella sp	21	6	350	0.0006
Arthrospira platensis	19	9	211	0.0123

NA: Not available.

Supplementary Table 7. Raw data used in geometric morphometric analysis

Diet	Gender	Individuals	Wings analyzed (R+L)
Asaia sp.	F	16	32
Asaia sp.	M	21	42
Escherichia coli	F	20	40
Escherichia coli	M	30	60
Tetramin ® Feed	F	17	34
Tetramin ® Feed	M	22	44
Saccharomyces cerevisiae	F	30	60
Saccharomyces cerevisiae	M	30	60
Pseudozyma sp.	F	29	58
Pseudozyma sp.	M	30	60
Tetramin ® Feed	F	23	46
Tetramin ® Feed	M	30	60

Supplementary Table 8. Scores of sample distribution test.

Non Normal Distribution		Normal Distribution			
M		F		M	
E	D	E	D	E	D
S. cerevisiae					
0.000004456	0.000001311	0.8169	0.9916	-	-
**Tetramin ®**					
0.03551	-	0.122	0.1387	-	0.0862
***Pseudozyma* sp.**					
0.0005214	0.0001664	0.5358	0.479	-	-
**Tetramin ®**					
0.005014	0.01314	0.6853	0.5239	-	-
***Asaia* sp.**					
0.02106	0.01062	0.4306	0.4903	-	-
E. coli					
-	-	0.2646	0.7693	0.3918	0.6024

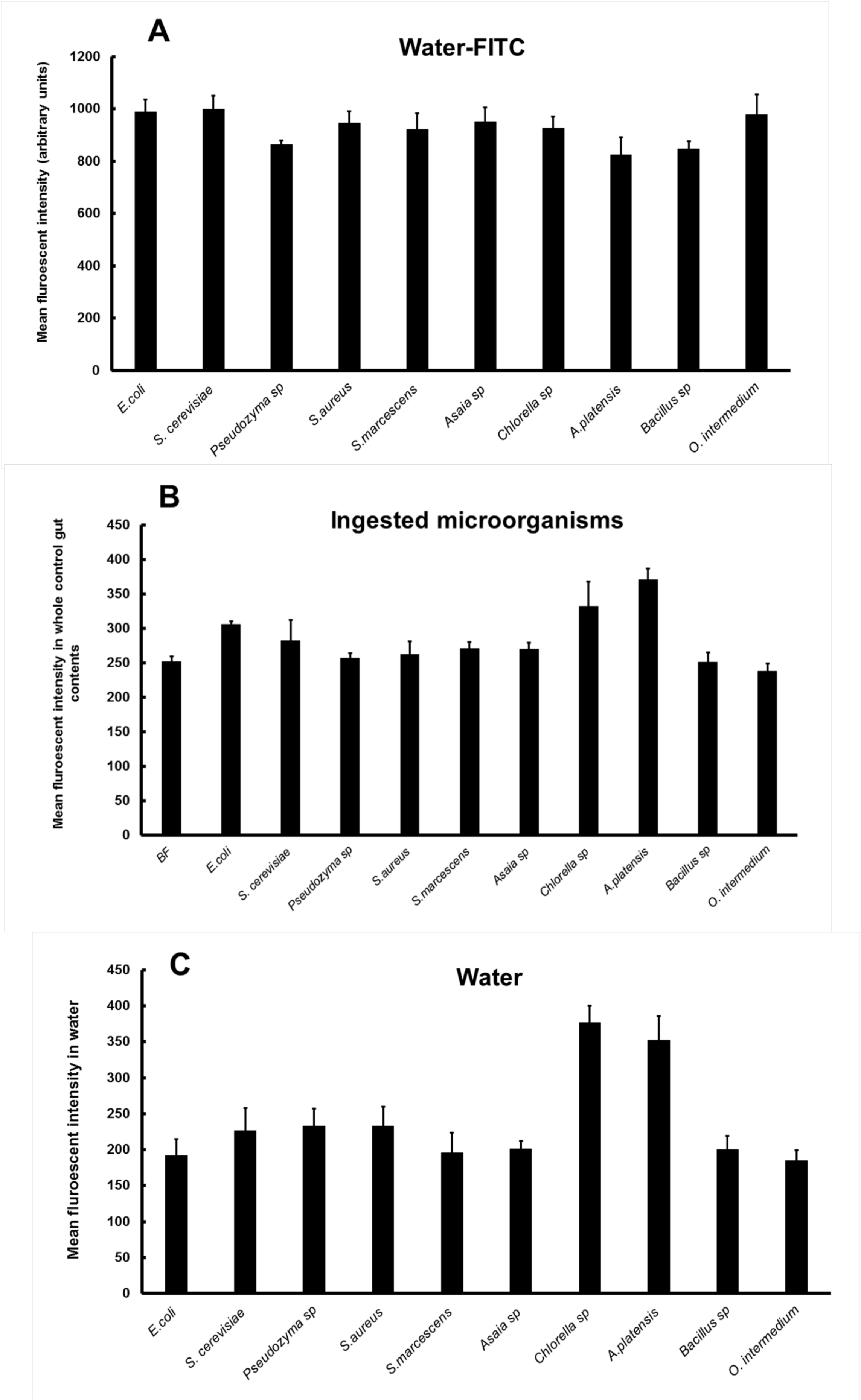

Figure S1. Mean fluorescent intensity of larval guts after 2 hours in **(A)** water with FITC-labeled microorganisms, **(B)** water with non FITC-labeled microorganisms (controls) and **(C)** standard rearing water.

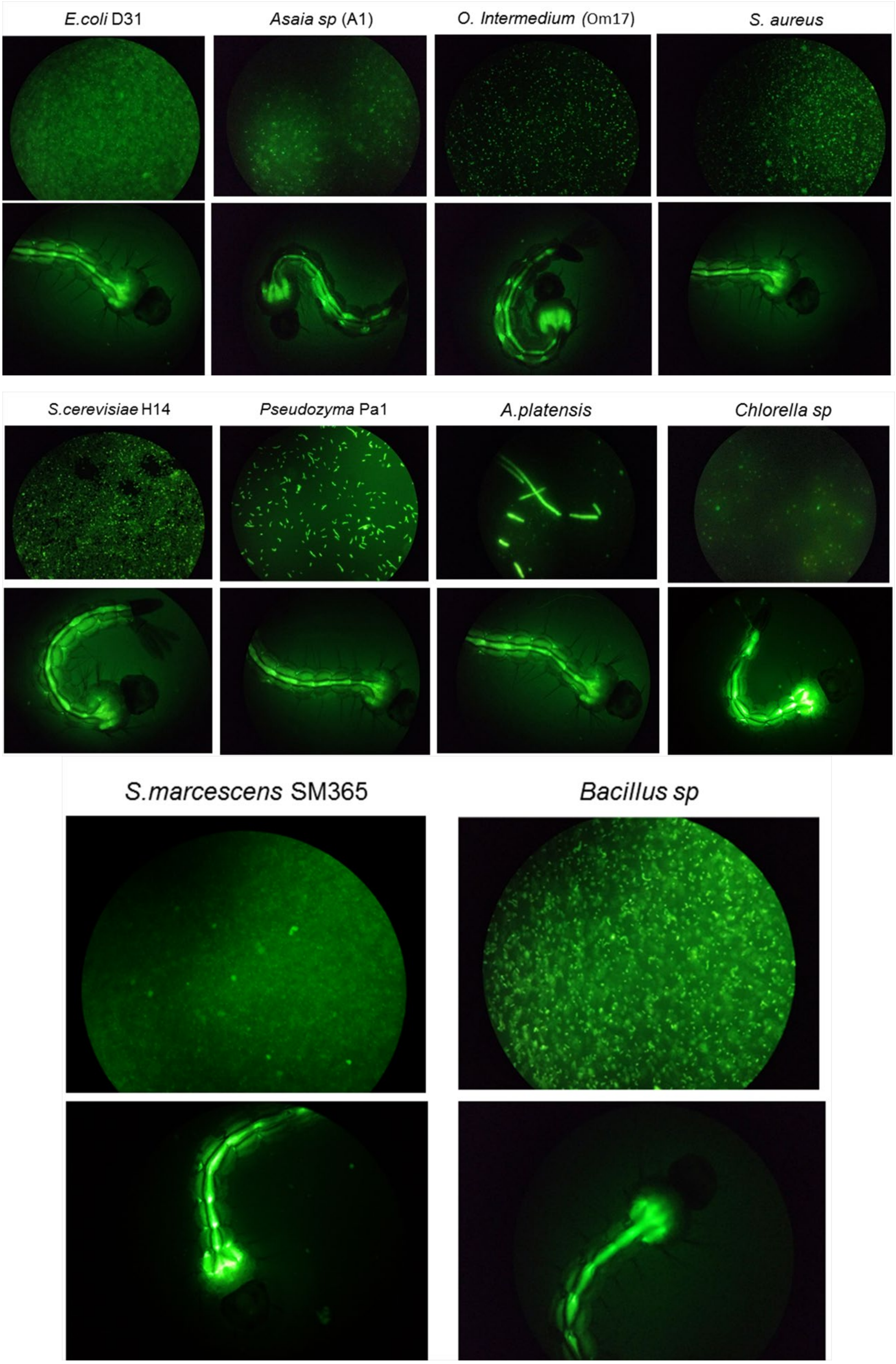

Figure S2. *Aedes aegypti* larvae consuming different microorganisms. Fluorescent images of each microorganism strain after FITC-labeling and individual larvae 2 hours after placing FITC-labeled microbial cells into rearing water. Note that fluorescent tracer is visible in the intestine of each larva 2 hours after exposure.
